# Reversal of aging-associated increase in myelopoiesis and expression of alarmins by angiotensin-(1–7)

**DOI:** 10.1038/s41598-023-29853-w

**Published:** 2023-02-13

**Authors:** Kishore Chittimalli, Jesmin Jahan, Anil Sakamuri, Hope Weyrick, Wink Winkle, Steven Adkins, Stefan W. Vetter, Yagna P. R. Jarajapu

**Affiliations:** 1 Department of Pharmaceutical Sciences, College of Health Professions, North Dakota State University, Sudro-16, Albrecht Blvd., Fargo, ND 58108 USA; 2 School of Biomedical Sciences, University of North Dakota, Grand Forks, ND 58202 USA

**Keywords:** Cell biology, Physiology

## Abstract

Aging is associated with chronic systemic inflammation largely due to increased myelopoiesis, which in turn increases risk for vascular disease. We have previously shown evidence for the therapeutic potential of Angiotensin-(1–7) (Ang-(1–7)) in reversing vasoreparative dysfunction in aging. This study tested the hypothesis that ischemic vascular repair in aging by Ang-(1–7) involves attenuation of myelopoietic potential in the bone marrow and decreased mobilization of inflammatory cells. Young or Old male mice of age 3–4 and 22–24 months, respectively, received Ang-(1–7) (1 µg/kg/min, s.c.) for four weeks. Myelopoiesis was evaluated in the bone marrow (BM) cells by carrying out the colony forming unit (CFU-GM) assay followed by flow cytometry of monocyte-macrophages. Expression of pro-myelopoietic factors and alarmins in the hematopoietic progenitor-enriched BM cells was evaluated. Hindlimb ischemia (HLI) was induced by femoral ligation, and mobilization of monocytes into the blood stream was determined. Blood flow recovery was monitored by Laser Doppler imaging and infiltration of inflammatory cells was evaluated by immunohistochemistry. BM cells from Old mice generated a higher number of monocytes (Ly6G^-^CD11b^+^Ly6C^hi^) and M1 macrophages (Ly6C^hi^F4/80^+^) compared to that of Young, which was reversed by Ang-(1–7). Gene expression of selected myelopoietic factors, alarmins (S100A8, S100A9, S100A14 and HMGb1) and the receptor for alarmins, RAGE, was higher in the Old hematopoietic progenitor-enriched BM cells compared to the Young. Increased expressions of these factors were decreased by Ang-(1–7). Ischemia-induced mobilization of monocytes was higher in Old mice with decreased blood flow recovery and increased infiltration of monocyte-macrophages compared to the Young, all of which were reversed by Ang-(1–7). Enhanced ischemic vascular repair by Ang-(1–7) in aging is largely by decreasing the generation and recruitment of inflammatory monocyte-macrophages to the areas of ischemic injury. This is associated with decreased alarmin signaling in the BM-hematopoietic progenitor cells.

## Introduction

Bone marrow resident cells are constantly mobilized into the blood stream in response to humoral and neuronal responses. Self-renewal of regenerative hematopoietic stem/progenitor cells (HSPCs) and generation of pro- and anti-inflammatory cells, myelopoiesis, is tightly balanced in physiological conditions. Endothelial progenitor cells (EPCs), a subpopulation of hematopoietic stem/progenitor cells, and monocyte-macrophage populations are known to be mobilized in response to vascular injury and participate in vascular regeneration^1–3^. There was no direct evidence for the differentiation of EPCs to endothelium but extensive evidence supports that these cells promote vascularization by paracrine mechanisms^4–6^. A consensus statement by Medina et al.^7^ on the nomenclature named these cells myeloid angiogenic cells (MACs).

In mice, monocytes expressing high levels of lymphocyte antigen 6C (Ly6C) have pro-inflammatory functions and express high levels of C–C chemokine receptor 2 (CCR2)^8^. Ly6C^+^ cells can migrate and differentiate into macrophages at the sites of injury or inflammation^9^. Two subsets of macrophages with distinct functions have been characterized, pro-inflammatory (F4/80^hi^Ly6C^hi^) and anti-inflammatory (F4/80^hi^Ly6C^low^) macrophages that were previously termed as M1 and M2 macrophages^10,11^. Clinical studies showed that circulating monocytes can predict cardiovascular incidents^12,13^. Monocyte-derived macrophages and dendritic cells were implicated in the etiology of hypertension^14,15^. Persistent increases in the circulating monocytes and tissue-resident macrophages increase the risk for the development or progression of cardiovascular disorders such as hypertension or ischemic vascular disorders^14,16^.

Aging is associated with a chronic inflammatory state with increased myelopoiesis in both humans and mice^17,18^. Impaired revascularization of ischemic areas with aging is largely due to elevated systemic and local vascular inflammation that can be attributed to the increased generation of myeloid-derived pro-inflammatory cells^19,20^. Kinetics of generation and mobilization of BM-resident cell populations is altered in pathological conditions including aging^21,22^. Bone marrow (BM)-resident HSCs derived from elderly subjects exhibited loss of quiescence and differentiation bias towards myelopoiesis with concurrent deficiency in the lymphopoiesis, which were not observed in the Young-HSC in ex vivo studies or in xenotransplantation models^18^. The bias was associated with transcriptional changes in the diverse subsets, notably, increased expression of myeloid-specification gene SELP and decreased lymphoid-specification genes SOX and FLT3, which was also observed in HSCs of aging mice^18,23,24^. Cell-intrinsic transcriptional changes occur in HSCs with each self-renewal and proliferation. The molecular mechanisms of proliferation-driven aging of HSCs in mice were documented, which include replicative stress, DNA damage, changes in the epigenetic landscape, and metabolic stress and changes in the niche environment^25,26^.

The renin angiotensin system (RAS) modulates both systemic and local inflammatory environments. The classical arm refers to angiotensin-converting enzyme (ACE)/Angiotensin II (Ang II)/AT1 receptor (AT1R) and the canonical pathway consists of cardiovascular protective ACE2/Ang-(1–7)/Mas receptor (MasR)^27^. Increased Ang II generation and AT1R expression in the vasculature with aging were reported^28,29^. Chronic suppression of Ang II by ACE-inhibitors or AT1R blockade ameliorated systemic inflammation and increased longevity^30,31^. In contrast, genetic ablation of ACE2 exacerbated vascular inflammation and atherosclerotic pathology^32^. MasR-deficiency increased pro-inflammatory expression in macrophages—IL6, iNOS, CCL2 and IL12 and stimulated migration to atherosclerotic plaques^33,34^.

Murine Lineage-negative, Sca-1^+^ and cKit^+^ (LSK) cells were consistently shown to be vasculogenic and accelerate vascular repair^35^. This population consists of HSPCs with ~ 10% stem cells^36^ and showed the highest potential of re-endothelialization. Importantly, LSK cells are mobilized in response to ischemic injury and accomplish revascularization via paracrine mechanisms^37,38^. Aging is associated with decreased number and impaired functions of vasculogenic progenitor cells, resulting in impaired revascularization of ischemic areas^39^. Alternatively, increased infiltration of inflammatory monocyte-macrophages impairs vascular regeneration following ischemic injury and increase tissue damage as shown in conditions such as diabetes^40–42^. We have previously shown that vasoreparative dysfunction in vasculogenic progenitor cells is accompanied with an imbalance in the ACE2/ACE activity that was negatively correlated with age^39^. Pharmacological treatment with Ang-(1–7) reversed mobilization-dysfunction in vasculogenic cells in response to ischemia in aging mice and restored blood flow recovery to ischemic areas^39^. The present study tested the hypothesis that ischemic vascular repair in aging by Ang-(1–7) involves attenuation of myelopoietic potential in the bone marrow and decreased recruitment of inflammatory cells to the sites of vascular injury. We further hypothesized that Ang-(1–7) modulates molecular expression of pro-myelopoietic factors in the BM-hematopoietic progenitor cells. We have determined relative ACE and ACE2 expressions BM-supernatants and AT1R and MasR expressions in the hematopoietic progenitor cells. Mobilization of inflammatory monocytes into the blood stream in response to ischemic injury was monitored and tissue-infiltration of monocyte-macrophages was examined. Myelopoiesis and the expression of pro-myelopoietic factors were determined in the hematopoietic progenitor-enriched BM cells. The S100 family of calcium-binding proteins and HMGb1, collectively known as alarmins, and the cognate receptor RAGE (receptor for advanced glycation end products)^43^ were evaluated by determining gene and protein expressions. HMGb1 is released passively upon cell damage or by active mechanisms triggered upon immune cell activation^44^. Extracellular HMGb1 mediates inflammation by enhancing differentiation of myeloid progenitor cells and by inducing migration, proliferation and differentiation of monocyte-macrophages via RAGE^45,46^.

## Results

**Figure 1:**
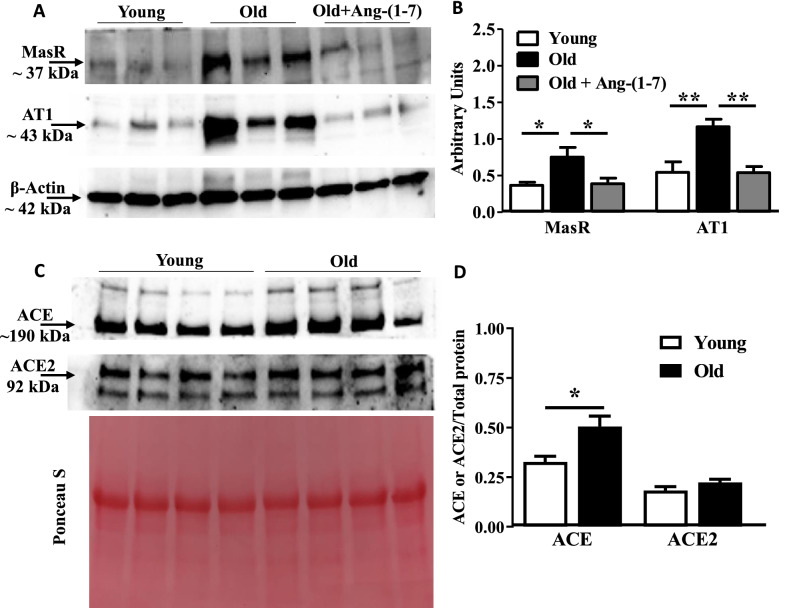
Aging alters expression of angiotensin receptors and ACEs in bone marrow cells: (**A**) Representative western blots for MasR and AT1R protein expressions in the bone marrow Lineage-depleted cells (**A**) and ACE and ACE2 (**C**) protein levels in the bone marrow supernatants derived from Young and Old mice. (**B**) Protein expression of both MasR and AT1R was higher in the Old cells compared to the Young, was decreased to levels similar to that observed in Young by Ang-(1–7) treatment (P < 0.05 and P < 0.01 for MasR and AT1R, respectively, n = 6). Groups were compared by One-way ANOVA followed by Tukey’s post-test. (**D**) ACE protein levels in the bone marrow supernatant were higher in the Old compared to the Young (P < 0.05, n = 8) while ACE2 levels were unchanged. Statistical differences were tested by using unpaired Students’ ‘t’-test.

MasR and AT1R expressions were first tested in the hematopoietic progenitor-enriched BM cells. Protein expression of both receptors were increased in Old cells compared to the Young (MasR—P < 0.05, AT1R—P < 0.01, n = 6) (Fig. 1A,B). Treatment with Ang-(1–7) decreased the expression in the Old cells to the levels similar to that observed in the Young (Fig. 1A,B). Protein levels of ACE and ACE2 were then determined by Western blotting in the BM-supernatants. ACE levels were higher in the Old compared to the Young BM-supernatants (P < 0.01, n = 6). No change was observed in ACE2 levels (Fig. 1C,D).

**Figure 2:**
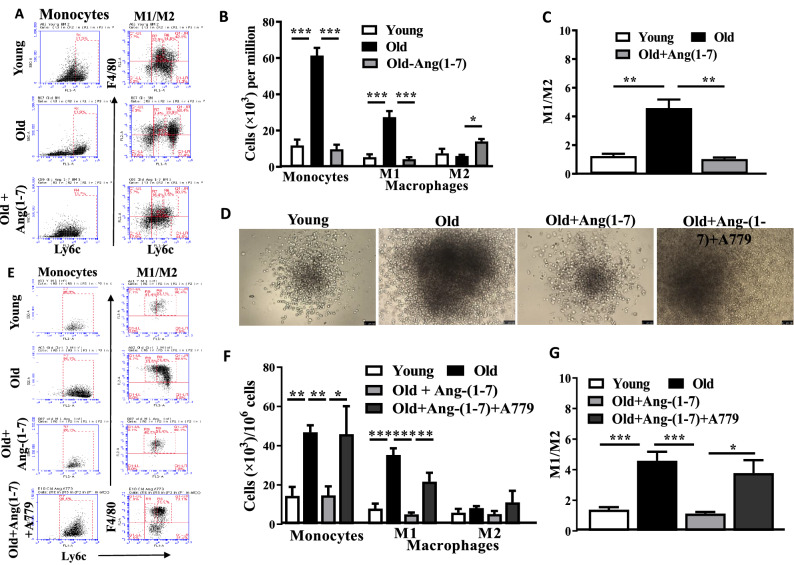
Myelopoiesis is increased in aging and reversed by Ang-(1–7): (**A**) Representative flow-cytometry dot plots for enumeration of monocyte-macrophages in the bone marrow derived from Young, Old or Old mice treated with Ang-(1–7). (**B**) Monocytes (Ly6G^-^CD11b^+^Ly6C^+^ cells) were significantly higher in the Old bone marrow compared to the Young (P < 0.001, n = 6) and decreased by Ang-(1–7) to the similar levels as Young (P < 0.001). M1 macrophages (Ly6G^-^CD11b^+^Ly6C^high^F4/80^+^) were higher in the Old bone marrow (P < 0.001) that was reversed by Ang-(1–7) (P < 0.001, n = 6). M2 macrophages (Ly6G^-^CD11b^+^Ly6C^low^F4/80^+^) in Old bone marrow were similar to that observed in the Young but significantly increased by Ang-(1–7) (P < 0.05, n = 6). (**C**) M1/M2 ratio was higher in the Old bone marrow (P < 0.01, n = 6) that was decreased by Ang-(1–7) (P < 0.01, n = 6). (**D**) Representative bright field images of CFUs derived from bone marrow cells of Young, Old or Old treated with Ang-(1–7) or Ang-(1–7) and A779. (**F**) Higher number of monocytes (P < 0.01, n = 6) and M1 macrophages (P < 0.001, n = 6) were generated in the CFU assay by Old bone marrow cells compared to the Young, that were decreased by Ang-(1–7) (P < 0.01 and P < 0.001 for monocytes and M1 macrophages, respectively, n = 6). Concurrent administration of A779 reversed the effect of Ang-(1–7) (P < 0.01 vs Ang-(1–7)-treated group). M2 macrophages were similar in all groups. (**G**) M1/M2 ratio is higher in CFUs generated by Old bone marrow cells (P < 0.001, n = 6) that was decreased by Ang-(1–7) (P < 0.001, n = 6). This effect was not observed with the simultaneous administration of A779 (P < 0.05 vs Ang-(1–7)-treated group). All groups were tested by One-way ANOVA with Tukey’s post-test for multiple comparisons. Scale bar measures 100 microns.

BM-resident monocytes, Ly6G^-^CD11b^+^Ly6C^+^ cells, were significantly higher in the Old BM compared to the Young (P < 0.001, n = 6) (Fig. 2A,B). In line with this, pro-inflammatory M1-macrophages, (Ly6G^-^CD11b^+^Ly6C^hi^F4/80^+^) were higher in the Old compared to the Young (P < 0.001, n = 6) but no significant difference was observed in the M2 macrophages (Ly6G^-^CD11b^+^Ly6C^lo^F4/80^+^) (Fig. 2A,B), which resulted in a higher M1/M2 ratio compared to the Young (P < 0.001, n = 6) (Fig. 2C). Ang-(1–7) treatment decreased the number of monocytes (P < 0.001 vs untreated, n = 6) (Fig. 2A,B) and reversed the imbalance in M1/M2 macrophages (P < 0.001, n = 6) compared to the untreated Old group (Fig. 2C). No change was observed in the number of M2 macrophages but the number of M1-macrophages were decreased in the Old group (P < 0.001 vs untreated Old) (Fig. 2B).

Then, we tested myelopoietic potential of BM-resident cells in vitro by CFU-GM assay. The total number of colony-forming units (CFUs) were similar in Young and Old BM however the size of the colonies was larger in cells derived from Old BM (Fig. 2D). The number of monocyte-macrophages in the colonies were characterized by dissociating to single-cell suspensions followed by flow cytometry. Cells from the Old group generated higher number of monocytes (P < 0.001, n = 6), and M1 macrophages (P < 0.001, n = 6) compared to the Young with similar number of M2 macrophages (Fig. 2E,F). M1/M2 (P < 0.001, n = 6) ratio was higher in the cells derived from Old BM compared to the Young (Fig. 2G). BM-cells derived from Ang-(1–7)-treated mice showed decreased myelopoiesis in BM-cells from Old mice as evident from the decreased number of monocytes compared to the untreated Old group and similar to that observed in the Young (P < 0.001, n = 6) (Fig. 2E,F) with a lower M1/M2 ratio (P < 0.001, n = 6) (Fig. 2G). Concurrent administration of the MasR antagonist, A779, reversed the effect of Ang-(1–7) on myelopoiesis in the Old group [monocytes (P < 0.05); M1 macrophages (P < 0.01) compared to the Ang-(1–7)-treated Old group (Fig. 2E–G)].

**Figure 3:**
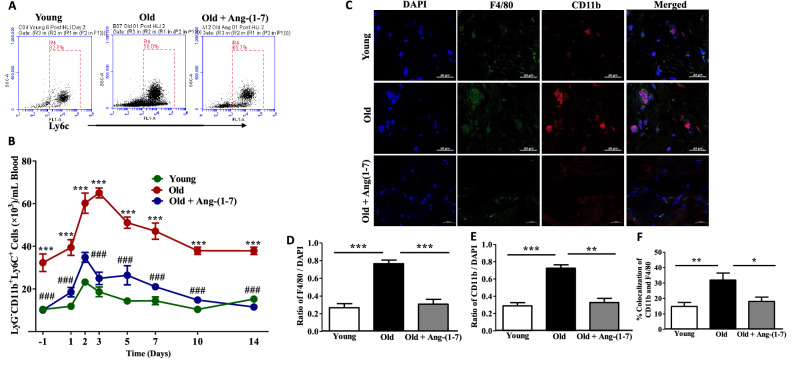
Mobilization and recruitment of monocyte-macrophages to areas of ischemia are higher in aging mice—Reversal by Ang-(1–7): (**A**) Shown were representative dot plots of monocytes (Ly6G^-^CD11b^+^Ly6C^+^ cells) from Young, Old and Ang-(1–7)-treated Old mice on Day-2 post-ischemic injury. (**B**) Time-course of mobilization of monocytes into the blood stream in three different experimental groups. Two-way ANOVA detected significant differences in the time course or mobilization in three groups (P < 0.0001, n = 6). Bonferrroni’s post-test for multiple comparisons identified monocyte levels in the circulation are higher in the Old group compared to Young at all time points (^***^P < 0.001, n = 6) and Ang-(1–7) treatment decreased the mobilization of monocytes in the Old group at all time points tested (^###^P < 0.001, n = 6). (**C**) Shown were representative immunofluorescence images of gastrocnemius muscle sections from ischemic limbs derived from three experimental groups stained for DAPI, F4/80 and CD11b for identifying monocytes (CD11b) and macrophages (F4/80). Scale bar measures 20 microns. (**D**,**E**) Quantification of CD11b and F4/80 fluorescence, respectively, in ischemic muscle sections from three different experimental groups. One-way ANOVA detected significant increase in macrophages (P < 0.001, n = 5) and monocytes (P < 0.001, n = 5) compared to the Young in the Old group, which was decreased by Ang-(1–7) (P < 0.001 and P < 0.01, respectively, n = 5) (two sections from each mouse).

In a different set of experiments mobilization and recruitment of inflammatory cells to the areas of ischemic injury were determined. Both Young and Old mice responded to HLI by increased mobilization of monocytes from BM into the blood circulation. In the Young group, peak mobilization was observed on day-2 by twofold, and gradually decreased to the pre-ischemic levels (n = 6). In the Old group, the number of monocytes in circulation was significantly higher, ~ threefold, compared to the Young. Further increase by threefold was observed in the Old group following HLI by day-3 and remained higher for fourteen days (P < 0.0001, Two-way ANOVA, n = 6) (Fig. 3A,B). Bonferroni post-test revealed that the increase was significant at all time points compared to the Young (P < 0.001). Our preliminary studies showed similar trends in the mobilization of monocytes in both male and female mice therefore further experiments were carried out in males. Treatment with Ang-(1–7) decreased the number of mobilized monocytes in response to HLI (P < 0.0001, Two-way ANOVA) (Fig. 3B). Bonferroni post-test identified that decrease in the circulating monocytes before and after HLI by Ang-(1–7) in the Old group was significant at all time points tested compared to the untreated Old group (P < 0.001). Then, we checked tissue-resident macrophages by immunocytochemistry. The CD11b^+^ or CD11b and F4/80 dual positive cells in the areas of injury were higher in the Old group compared to the Young, which were decreased by Ang-(1–7) treatment (P < 0.001, n = 6) (Fig. 3C–F).

**Figure 4:**
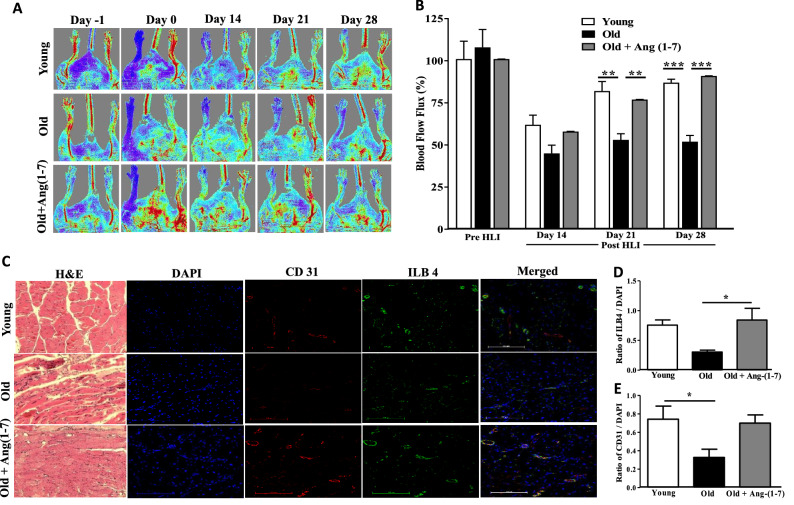
Blood flow recovery and vascularization in the areas of ischemia were lower in the Old that was restored by Ang-(1–7): (**A**) Shown were representative pseudo-color images of blood flow obtained by Laser Doppler imaging in different treatment groups before (day -1) and at day-0, day-14 and day-28 after HLI. (**B**) Blood flow recovery was lower in the Old group compared to the Young on day-21 an day-28, which was restored by Ang-(1–7) treatment. P < 0.01 and ***P < 0.001, n = 6. Two-way ANOVA with Bonferroni detected significant differences among treatment groups (P < 0.0001). Bonferroni post-test was used to perform multiple comparisons. (**C**) Shown were representative bright field images of Hematoxylin and Eosin (H&E) staining and fluorescence images with CD31 and isolectin-B4 (IlB4) staining of gastrocnemius muscle sections. (**D**,**E**) Intensity of capillary density with CD31 or IlB4-positivity was quantified by using ImageJ software. Capillary density determined by either CD31 or IlB4 was lower in the ischemic areas derived from Old mice compared to the Young, which was increased by Ang-(1–7) treatment. *P < 0.05, One-way ANOVA with Tukey’s post-test for multiple comparisons (n = 6).

In agreement with our previous study^39^, blood flow was progressively recovered following HLI in the Young mice with the maximum blood flow of 88 ± 4% compared to that found in the nonischemic contralateral limb by day-28 following ischemic injury. Maximum blood flow recovery in the Old group at day-28 post-HLI was lower compared to the Young (51 ± 7%, P < 0.001, n = 6) (Fig. 4A,B). In the group of Old saline-treated mice, either toe or foot amputations were observed. Blood flow recovery was increased by treatment with Ang-(1–7) to 84 ± 5% (n = 6) with no evidence for amputations (Fig. 4A,B). In line with blood flow recovery, H&E staining revealed presence of necrotic areas in the gastrocnemius muscle derived from Old mice compared to the Young (Fig. 4C), while muscle sections derived from Ang-(1–7)-treated Old mice showed no signs of necrosis (n = 6, Fig. 4C). Along similar lines, the capillary density as measured by CD31 or IlB4 positivity, was lower in the Old group compared to the Young in the ischemic regions of gastrocnemius muscle (P < 0.05, n = 6), which was significantly increased by Ang-(1–7) treatment (P < 0.05, n = 6) (Fig. 4C–E).

**Figure 5:**
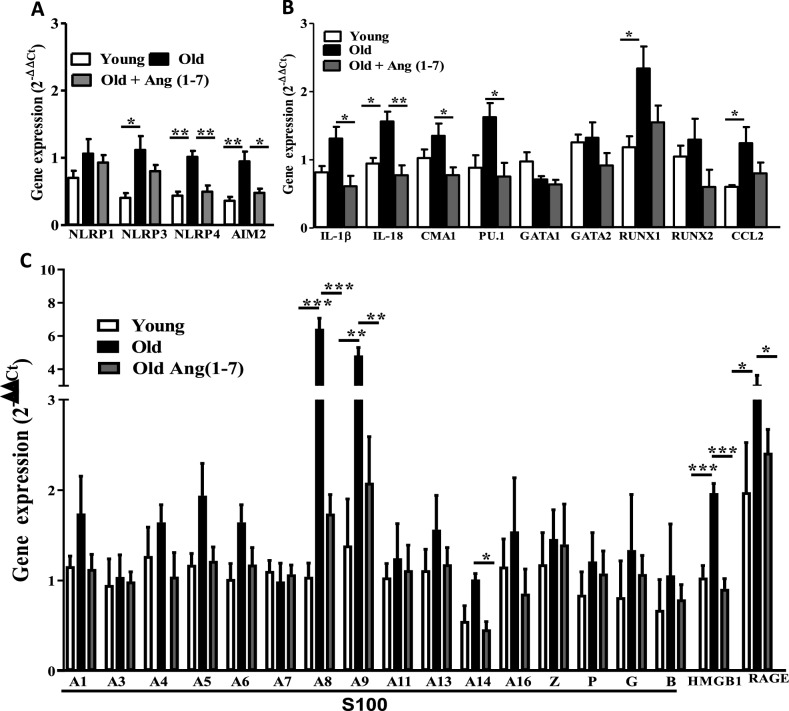
Expression of alarmins in the aging bone marrow cells and the effect of Ang-(1–7). (**A**,**B**) Gene expression of selected pro-myelopoietic and pro-inflammatory factors were altered in aging and by Ang-(1–7). *P < 0.05 and **P < 0.01, n = 5. One-way ANOVA with Tukey’s test for multiple comparisons was used to test for statistical significance. (**C**) Gene expression of alarmins. S100A8, S100A9, S100A14 and HMGB1, and the receptor RAGE were higher in the bone marrow cells of Old mice compared to the Young (*P < 0.05, **P < 0.01, ***P < 0.001, n = 5) and it was reversed by Ang-(1–7) compared to the untreated (*P < 0.05, **P < 0.01, ***P < 0.001, n = 5). Groups were compared by One-way ANOVA followed by Tukey’s post-test for multiple comparisons.

Then, the study focused on characterizing molecular signatures of increased myelopoiesis in Old BM hematopoietic progenitor-enriched cells. Gene expression, of pro-myelopoietic and pro-inflammatory factors, and receptors for alarmins revealed significant increase in the Old BM cells that were decreased by Ang-(1–7) treatment. Expression of NLRP3 (P < 0.05, n = 5), NLRP4 and AIM2 was increased in Old cells compared to the Young (P < 0.01, n = 5) (Fig. 5A). Increase in NLRP4 or AIM2 expression was reversed by Ang-(1–7) (P < 0.01 and P < 0.05, respectively, n = 5) but the decrease in NLRP3 did not achieve statistical significance. GATA1, GATA2 and RUNX2 expressions were unaltered in Old cells (Fig. 5B). IL-18, RUNX1, CCL2 expressions were increased in Old compared to Young cells (Fig. 5B). Ang-(1–7) decreased IL-18 in the Old (P < 0.01, n = 5) (Fig. 5B). Expression of IL-1β, CMA1 or PU.1 was increased in Old cells but did not achieve significance, however a statistically significant decrease was observed following treatment with Ang-(1–7) (P < 0.01, n = 5) (Fig. 5B). Among several alarmins that were tested, expression of S100A8 (P < 0.001), S100A9 (P < 0.01), S100A14 (P < 0.05) and HMGb1 (P < 0.001) were increased in Old cells, all of which were decreased by Ang-(1–7) (P < 0.05–0.001, n = 5) (Fig. 5C). Furthermore, expression of the receptor for alarmins, receptor for advanced glycation end products (RAGE), was increased in Old cells and that was decreased upon Ang-(1–7) treatment (P < 0.001, n = 5) (Fig. 5C).

**Figure 6:**
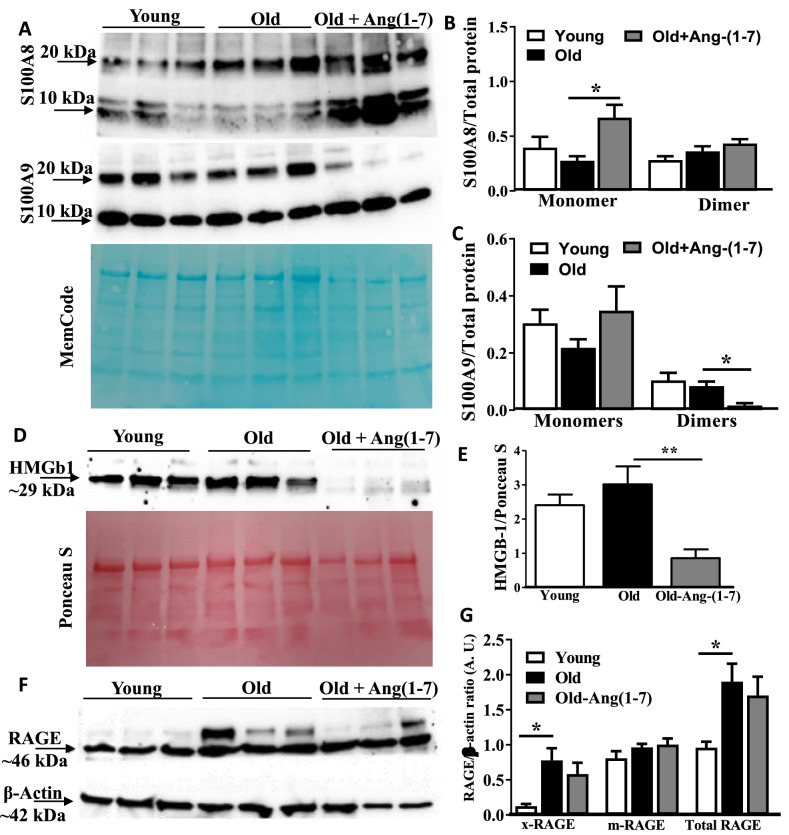
Protein levels of selected alarmins in the bone marrow supernatants derived from aging mice and the effect of Ang-(1–7): (**A**) representative western blots for S100A8 and S100A9 in the bone marrow supernatants from Young, Old and Ang-(1–7)-treated Old mice. Memcode was used to estimate the total protein. (**B**) S100A8 monomers were similar in Young and Old groups, Ang-(1–7) increased the levels in the Old compared to the untreated Old group (*P < 0.05, n = 6). No significant differences were observed in dimer levels. (**C**) S100A9 monomers were similar in all three groups. On the other hand, dimer levels were similar in the Young and Old but Ang-(1–7) decreased the dimer levels in the Old group (*P < 0.05, n = 6). (**D**,**E**) Representative western blots of HMGB1 in three different groups of bone marrow supernatants and Ponceau S was used for the estimation of total protein. HMGB1 levels were similar in Young and Old groups. Ang-(1–7) decreased the protein in the Old group (**P < 0.01, n = 6). (**F**,**G**) Representative western blots of RAGE in the bone marrow cells derived from three experimental groups with β-actin as an internal control. While the protein levels of mRAGE were similar in all three groups, xRAGE and total RAGE were higher in the Old bone marrow cells compared to the Young (*P < 0.05, n = 6). Ang-(1–7) has no effect on RAGE levels in Old group. One-way ANOVA was used for testing the statistical differences among experimental groups for comparing protein levels.

Protein levels of selected factors were analyzed in the BM-supernatants. S100A8 and S100A9 protein levels were differentially altered in BM-supernatants derived from Old mice. Unexpectedly, two bands were observed for S100A8 and S100A9 in the western blots with approximate molecular weights of about 10 and 20 kDa. According to the manufacturer, the antibody used for S100A8 detection does not cross-react with S100A9 and the S100A9 antibody does cross-react with S100A8. We therefore believe that both bands represent S100A8 and S100A9 expression levels, respectively. A possible explanation for the 20 kDa band is the formation of a zinc containing homo or hetero-dimer of S100A8 and S100A9, which could be stable under the conditions used for electrophoresis. Overall, S100A8 expression was lower in the Old compared to the Young and was increased by Ang-(1–7) (P < 0.05, n = 6) (Fig. 6A,B). Expression levels of S100A9 appeared to be similar in both Young and Old groups (Fig. 6A–C). HMGb1 levels were similar in Young and Old bone marrow supernatants and a significant decrease was observed by Ang-(1–7) (P < 0.01, n = 6) (Fig. 6D,E). Then, protein expression of RAGE by western blotting revealed a band in all samples with an apparent molecular weight of 46 kDa, which can be attributed to the non-glycosylated form of RAGE. A second band was observed with a higher molecular weight in particular in the samples derived from Old mice. This band is attributed to a glycosylated form of RAGE (Fig. 6F,G). RAGE is well known to have multiple glycosylated forms, which electrophoretically migrate differently than the non-glycosylated forms and named xRAGE and mRAGE, previously^47,48^. Expression of xRAGE was higher in the Old cells compared to the Young. Similar trend was observed in the total RAGE. However, Ang-(17) treatment had no impact on xRAGE or total expression, in contrast to the observations in the gene expression (Fig. 6F,G).

**Figure 7:**
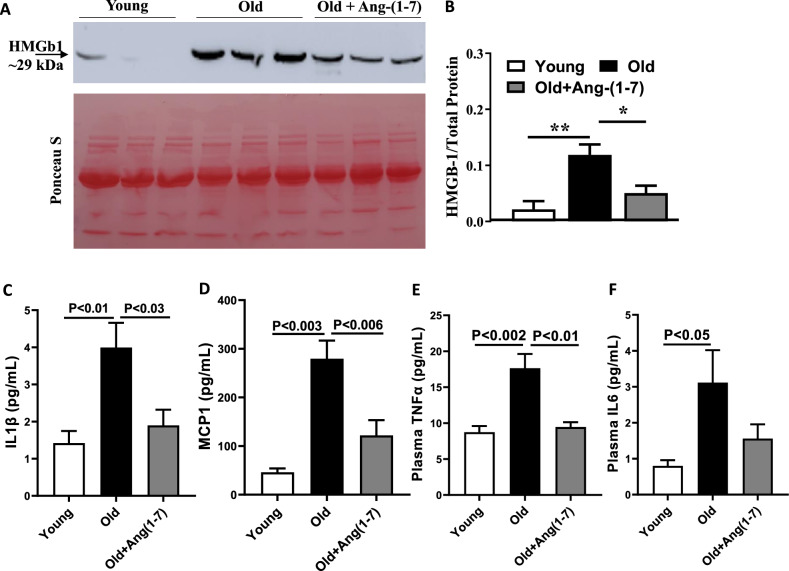
Ang-(1–7) decreased the concentrations of circulating inflammatory factors in the aging mice. (**A**) Representative western blots of HMGB1 levels in the plasma samples of Young, Old and Ang-(1–7)-treated Old mice. Ponceau S was used for the estimation of total protein. (**C**–**F**) Old mice have higher levels of HMGB1 in the circulation (**P < 0.01, n = 6) that were decreased by Ang-(1–7) (*P < 0.05, n = 6). IL1β, MCP1, TNFα and IL6 levels were higher in the plasma samples of Old mice compared to the Young. With exception of IL6, Ang-(1–7) decreased the levels of pro-inflammatory factors in the Old mice (n = 6).

Lastly, selected pro-inflammatory factors in the circulation were analyzed in all three experimental groups. Plasma levels of HMGb1, analyzed by western blotting, were higher in the Old mice compared to the Young (P < 0.01, n = 6) and were significantly decreased by Ang-(1–7) treatment (Fig. 7A,B). Similar trend was observed in the circulating levels of IL1β, MCP1, and TNFα in the Old that were analyzed by Luminex multiplex bead assay. Increased levels of these factors in the Old group were decreased by Ang-(1–7) (Fig. 7C–E). While significant increase was observed in IL6 levels in the Old compared to the Young (P < 0.05, n = 6), decrease by Ang-(1–7) did not achieve statistical significance (Fig. 7F).

## Discussion

This study reports for the first-time pharmacology of Ang-(1–7) in the aging bone marrow inflammation with novel findings. Aging BM-HSPCs have increased expression of MasR as well as AT1R with increased soluble ACE in the BM-supernatants. Increased myelopoietic potential in the aging BM accompanied higher expression of alarmins, and RAGE, in the hematopoietic progenitor cells. Systemic inflammation in aging was decreased by Ang-(1–7). Decreased myelopoiesis in the aging bone marrow cells by Ang-(1–7) resulted in decreased mobilization and recruitment of monocyte-macrophages to the areas of ischemia were higher in the Old that were attenuated by Ang-(1–7) resulting in the restoration of blood flow and vascularization. MACs and monocyte-macrophages mobilized and recruited to the areas of ischemia play an important role in accomplishing revascularization^7,49^ however increased numbers of pro-inflammatory cells and elevated levels of circulating pro-inflammatory factors in aging, attenuate this response. Although our previous study reported impaired mobilization of LSK cells in response to ischemic injury, age-dependent changes in the immunophenotypic functions of MACs were not evaluated.

Our study showed increased expression of both AT1R and MasR in the Old BM-cells suggesting actions of both Ang II and Ang-(1–7) would be elevated. While ACE levels are increased in the BM-microenvironment, ACE2 levels were unchanged with age. This likely increases ACE-dependent Ang II generation with relatively lower generation of ACE2-dependent Ang-(1–7) levels, although the peptides were not analyzed in the current study. Increased levels of soluble ACE is most likely due to the shedding of ACE. Previous study showed increased ACE with concurrent decrease in ACE2 expression in Old-HSPCs^39^ and increased ACE expression is likely to be associated with elevated shedding of ACE protein fragment that retains catalytic activity^50^. While shedding of ACE was previously shown in disease conditions^51–53^, this is the first observation in an experimental model of aging and requires further investigation to characterize this phenomenon in other tissues and physiological significance in aging. Several lines of evidence support pro-myelopoietic functions of Ang II/AT1R in BM cells. Monocyte-macrophage generation and vascular inflammation that increase risk for hypertension or aneurysm, were shown to be increased via Ang II/AT1R^54^. Ang II induced myelopoiesis and macrophage polarization with increased M1 and decreased M2 macrophages thus promoting systemic inflammation^55,56^. ACE- or AT1R-deficient BM cells increased macrophage influx, aortic inflammation and atherosclerosis in a diet-induced model while ACE2-deficient-BM cells exacerbated the lesions^57–59^. However, Ang-(1–7) treatment interestingly decreased the expression of both AT1R or MasR in the old BM cells to levels similar to that in the Young. Therefore, increased MasR expression is Old-HSPCs is likely due to decreased levels of agonist, Ang-(1–7) while decrease in AT1R could be mediated by MasR activation.

MasR activation decreased myelopoiesis, generation of monocyte-macrophages in the aging bone marrow. Old mice have increased number of inflammatory cells in the circulation that were further increased following ischemia, likely by mobilization from bone marrow into the blood stream. In agreement with this, selected pro-inflammatory factors are elevated in the circulation. Previous studies have shown mobilization and recruitment of monocytes to areas of ischemia in support of their participation in the vascular regeneration^60–62^ however chronic elevation of systemic and local inflammation indeed oppose vascular regeneration. Elevated levels pro-inflammatory factors induce apoptosis and autophagy in the microvascular endothelium and in the BM-derived vasculogenic progenitor cells thus attenuating their reparative potential^63–66^. Our study for the first time showed the time-course of mobilization, which parallels the mobilization kinetics of vasoreparative progenitor cells, LSK cells, in the young as shown before^39,67^. Monocytes recruited to ischemic areas differentiate to macrophages, which were also shown to participate in resolving inflammation and accelerating vascular regeneration. However, the number of monocyte-macrophages in the ischemic areas were higher in the Old group compared to the Young and overall blood flow recovery was lower that accompanied tissue necrosis and impaired vascular regeneration, which can be attributed to the exaggerated pro-inflammatory environment. Mobilization and recruitment of Ly6C^hi^ monocytes to areas of ischemia exacerbate local inflammation and proteolytic activity impair revascularization^68,69^. Increased infiltration and prolonged accumulation of Ly6C^hi^ cells and delayed transition to anti-inflammatory M2 macrophages impair vascular reendothelialization and blood flow recovery^70,71^. Ang-(1–7) by decreasing the generation of inflammatory monocyte-macrophages in bone marrow, reduced the number of cells in the circulation and infiltration to ischemic areas and enhanced transition of monocytes to Ly6C^lo^ macrophages, which collectively decreased systemic and local inflammatory stress that restored ischemic vascular repair.

HSPCs provide impetus for vascular regeneration largely via paracrine mechanisms^5,60^. Here we show altered gene expression profile with pro-inflammatory switch with increased IL1b, IL18, S100A8, S100A9 and HMGb1, that collectively oppose angiogenic functions. This is further supported by the inhibitory effect of RAGE antagonist on monocyte-macrophage generation in Old BM cells suggesting exaggerated myelopoiesis in aging was largely mediated by autocrine/paracrine activation of RAGE. Inflammaging is increased production of pro-inflammatory cytokines and chemokines systemically and locally in BM with aging and expression of receptors for pro-inflammatory factors in BM-stem/progenitors^72,73^. Our data showed evidence for increased expression of genes indicative of myelopoietic upregulation of pro-inflammatory signaling in the Old BM-cells and increased levels of selected factors in the circulation, which were reversed by Ang-(1–7).

The expression of RAGE and RAGE ligands S100A8, S100A9, S100A14 and HMGb1, were increased in BM-resident stem/progenitors of aging mice that was reversed by Ang-(1–7). RAGE is a member of superfamily of immunoglobulin receptors and exist in both membrane-bound and soluble form lacking transmembrane domain^43,74^. The antibody that we have used to detect RAGE revealed the presence of two isoforms of membrane-bound receptor in the cell lysates, which are attributed to differentially glycosylated forms of RAGE^47^. Compared to the Young BM cells, cells from the Old mice showed higher expression of glycosylated RAGE that were decreased by Ang-(1–7). S100A14 expression was quite low compared to the others therefore protein levels of S100A8, S100A9 and HMGb1 in the bone marrow environment were determined. Although, changes were observed in either dimer or monomer levels of S100A8 or S100A9, HMGb1 levels were apparently higher in Old BM-cells. The HMGb1-RAGE signaling axis has been shown to mediate angiotensin II (Ang II)-induced endothelial dysfunction that was attenuated by disruption of the HMGb1/RAGE axis^75^. NFκB is the downstream signaling molecule that mediates gene transcription of pro-inflammatory factors by RAGE activation and is ERK1/2-dependent^76,77^. Alarmins, S100A8/A9, derived from BM-cells including monocyte-macrophages were hypothesized to promote myelopoiesis in bone marrow^78,79^. The most stable and biologically relevant form of S100A8/A9 is a heterodimeric complex, which is formed after release^80^. Receptor Binding capacity is in the increasing order of S100A8 ≤ S100A9 ≤ S100A8/A9, the heterodimer, calprotectin, with highest receptor binding affinity^81^ and more effective in inducing proinflammatory molecules IL-6, IDAM-1, VCAM1 and MCP1 largely via TLR4 and RAGE^82,83^. Importantly, S100A9 homodimers or S100A8/A9 heterodimers effectively induce CD11b expression/affinity in monocytes and influence their migration^84,85^. Our data indicate that S100A9 dimers, and HMGb1 that were clearly decreased by Ang-(1–7) suggest that these two alarmins and the receptor significantly contribute to the beneficial effects of Ang-(1–7) in the Old mice. The effects of Ang-(1–7) on S100A9 dimers, HMGb1 and xRAGE were unambiguous. Experiments with RAGE-inhibitor further confirmed that autocrine/paracrine activation of RAGE indeed mediated pro-myelopoietic functions of Old BM-cells.

**Figure 8:**
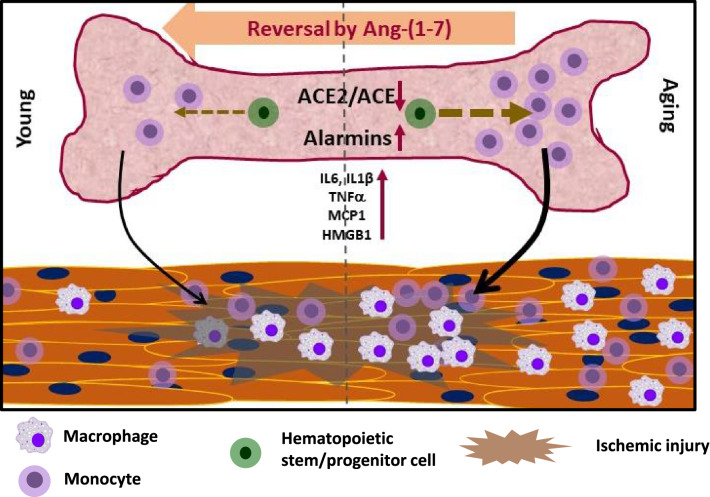
Summary of findings. Aging in mice is associated with imbalance in ACE2/ACE in the BM-environment and increased myelopoiesis and alarmin expression in the hematopoietic progenitor cells. Increased generation of monocyte-macrophages resulted in systemic inflammation and tissue infiltration of monocyte-macrophages. Recruitment of macrophages to areas of ischemic injury was higher in the Old mice, which resulted in impaired recovery. Ang-(1–7) via activation of MasR reversed aging-associated changes in alarmin expression, myelopoiesis and recruitment of monocyte-macrophages following ischemic insult.

In conclusion, aging is associated with systemic inflammation largely due to increased generation of pro-inflammatory monocyte-macrophages, which in turn impairs vascular regeneration following ischemic injury. Apart from increased expression of pro-myelopoietic factors, alarmins S100A8, S100A9, HMGB1 and the receptor RAGE are highly expressed in aging mouse bone marrow. Activation of MasR in the aging BM-cells by Ang-(1–7) decreased myelopoiesis, alarmin expression and circulating pro-inflammatory cells and factors. Mobilization and recruitment of monocyte-macrophages to sites of ischemic injury was higher in aging and blood flow recovery and revascularization were lower. These dysfunctions were reversed by Ang-(1–7) and accelerated healing following ischemic injury in aging. Therefore, MasR activation is a promising approach for reversing aging-associated inflammation and vascular disease. Findings are summarized in the Fig. 8.

## Limitations and future directions

While the study reports novel findings, it is not free from limitations. We have carried out a few pilot experiments to find out major differences in the sex-dependent differences in the mobilization of monocytes to ischemic injury, all sets of experiments were carried out in male mice. This represents a major limitation of the study. Gene expression profiling was carried out in enriched hematopoietic progenitor cells, which is a heterogeneous population. Despite the heterogeneity, gene expression of myelopoietic factors is clearly increased, which agrees with results from CFU-GM assays. However, sorted cell populations of different progenitor subsets were not studied for gene expression, which is a limitation. Along similar lines, characterization of the complete landscape of EPC phenotype and contribution of MACs to the impaired vascularization would enhance our understanding of age-associated impairments in the ischemic vascular repair.

This study created novel avenues for further research mainly for understanding molecular mechanisms of myelopoiesis and the pharmacological potential of vasoprotective axis of RAS. While the study evaluated the beneficial effects of Ang-(1–7)/MasR pathway, the recently identified Alamandine/Mas-related G-protein couple receptor, D (MrgD) axis requires systematic investigations. Authors are particularly interested in determining how metabolic stress in aging HSCs can be modulated by angiotensins, which would help designing novel pharmacotherapeutic strategies for reversing hematopoietic imbalance and impaired vasculogenesis in aging.

## Methods

### Animal model and treatments

All animal studies were approved by the Institutional Animal Care and Use Committee at North Dakota State University (NDSU). All authors complied with and all methods are reported in accordance with ARRIVE guidelines. All methods were carried out in accordance with relevant guidelines and regulations.

Male C57BL/6 mice at Young age (12–14 weeks) or Old (22–24 months)^86^, were used in the study. Mice were maintained on a 12-h light–dark cycle with food and water ad libitum. Ang-(1–7) or A779 was administered as a continuous infusion by subcutaneous osmotic pumps at the infusion rate of 1 μg/kg/min for four weeks as described^39^. Two cohorts of mice were used in the study. One cohort of mice was subjected to hind-limb ischemia and another cohort of mice was used for obtaining BM supernatants and peripheral blood samples. BM supernatants were collected by harvesting the whole bone marrow from two femurs and tibiae of each mouse in PBS. The BM suspensions were centrifuged at 1200 g and the cell-free supernatants were collected and concentrated to 0.5 mL by using Amicon centrifugal filters (3 kDa molecular weight cutoff) (Millipore Sigma). Supernatants were preserved at − 80 °C for analysis.

Hind–limb Ischemia (HLI) was induced in mice by femoral artery ligation followed by excision as described before^67^. Blood-flow in the hind-limbs was measured by imaging the flux (blood × area^−1^ × time^−1^) by using a Laser Doppler imaging system (Moor Instruments Inc.) under isoflurane anesthesia and was reported relative to the mean blood flux in the contralateral non-ischemic limb.

### Isolation of bone marrow cells

Mononuclear cells (MNCs) were isolated from bone marrow (BM) cell suspension by density gradient centrifugation using Ficoll (GE Healthcare). Layer of MNCs was separated and used for enrichment of lineage-depleted cells or for the enumeration of vasculogenic progenitor cells and monocyte-macrophages as described below. Hematopoietic progenitor cells were enriched by negative selection by using immunomagnetic enrichment kit (EasySep, Cat#19856A, Stemcell Technologies Inc.) as per manufacturer’s instructions. Suspension of MNCs was incubated with a cocktail of antibodies for CD5, CD11b, CD19, CD45R, 7–4, Ly-6G/C (Gr-1), and TER119. Then, the cells were labeled by Tetrameric Antibody Complexes that recognize biotin and dextran-coated magnetic particles and antibody-bound cells were then separated by using EasySep magnet to obtain enriched hematopoietic progenitor cells. These cells were used for gene expression analysis.

### Flow cytometry

Peripheral blood samples were collected from submandibular vein and white blood cells were isolated after treatment with red blood cell lysis buffer. Cells were suspended in 100 µL of cell staining buffer (Biolegend) and incubated for 5 min with 0.5 µL of Trustain (Biolegend). Later, the cells were incubated with fluorescent conjugated antibodies at 4 °C for 45 min. For enumeration of inflammatory cells Ly6G-PE CF594 (BD Bioscience), CD11b-APC (Invitrogen), Ly6C-FITC (BD Biosciences) and F4/80-PE (Biolegend) antibodies were used. Dead cells were identified by using 7AAD (BD Pharmingen). Flow cytometry was carried out by using Accuri C6 (BD Biosciences) cytometer by following standard protocols as per the gating strategy shown in the Supplementary Fig. S1 for the enumeration of monocytes (Ly6G^-^CD11b^+^Ly6C^hi^), and M1 (F4/80^+^Ly6C^hi^) or M2 (F4/80^+^Ly6C^lo^)^40,42^.

### Clonogenic assay

BM-MNCs were obtained by using Ficoll reagent and either ten or five thousand cells were tested for CFU-GM assay in a 30 mm culture dish by using Methocult medium (Methocult GF M3534, Stem Cell Technologies) as per manufacturer’s protocol. After 10 days, colonies were counted, and bright field images were taken by using a microscope (Leica). Then, colonies were dissociated, and inflammatory cells were enumerated by flow cytometry as described above. Where applicable, antagonist or vehicle was added to the cell-suspension prior to mixing with Methocult.

### Western blotting

Protein samples from BM supernatants or plasma or from lysates of hematopoietic progenitor-enriched BM-cells from different groups were assayed by using BCA assay kit (Thermo scientific pierce, Cat# 23225). Protein samples were boiled at 95 °C for five minutes in Laemmli buffer containing β-mercaptoethanol and snap-cooled and separated by electrophoresis using 4–20% SDS–PAGE gradient gels (Bio-Rad). Proteins were transferred to PVDF membranes and blocked for one hour in 2% BSA in TBST. Later, membranes were incubated with a primary antibody for overnight at 4˚C. Blots were then washed with TBST followed by incubation with an appropriate secondary antibody tagged with horse radish peroxidase (1:3000 dilution) at room temperature for two hours. Blots were washed and protein bands were detected using enhanced chemiluminescence substrate (Pierce ECL substrate). β-actin was used as an internal control for western blotting of proteins derived from cell lysates. Ponceau S or Memcode was used to quantify the total protein in the BM-supernatants or plasma. The intensities of the protein bands were quantified by using ImageJ software (https://imagej.nih.gov/ij/)^87^. The list of antibodies with concentrations is provided in the Supplementary Table S1. Original western blot images are included in the online Supplementary data S1.

### Semi-quantitative real-time polymerase chain reaction (RT-PCR)

RNA was extracted from frozen cells by using Trizol. The concentration and purity of RNA were determined by a spectrophotometer (NanoDrop Technologies). RNA (1 µg) was reverse-transcribed by using a qScript cDNA synthesis kit (Bio‐Rad) according to the manufacturer's protocol. For the detection of DNA synthesis in real-time, SYBR green was used. Each sample contained 50 ng DNA, 10 μM of forward and reverse primers, and the iQ SYBR green containing supermix (Bio‐Rad). Primer sequences were provided in the resources table. β-actin was used as an internal housekeeping gene. The reactions were run in the Quantitative PCR System (Stratagene Mx3000P) using the following conditions: 3 min at 95 °C, followed by 40 cycles of 10 s at 95 °C (denaturation step), 30 s at 55 °C (annealing step), and 30 s at 72 °C (extension step). Primer sequences with concentrations were listed in the Supplementary Table S2.

### Immunohistochemistry

Mouse gastrocnemius muscle specimens were isolated and fixed in 4% Paraformaldehyde solution for 24 h at room temperature, which were then embedded in paraffin blocks to obtain sections of 5 microns thickness. These sections were deparaffinized using xylene then hydrated and stained with Harris Hematoxylin solution and Eosin Y Phloxine B solution (Electron Microscopy Sciences). Sections mounted on glass slides by using DAPI (Vector laboratories) for staining nuclei and Permount (Electron Microscopy Sciences) for imaging by a Fluorescence microscope (Leica).

For immunofluorescence staining, deparaffinized sections were subjected to epitope retrieval procedure by steaming in sodium citrate buffer (pH 6.0). Then, normal goat serum was used to avoid non-specific binding prior to incubation with primary and secondary antibodies by using standard protocols. CD31 and IlB4 were used for determining vascular density. CD11b and F4/80 antibodies were used to detect monocyte-macrophages. The list of primary and secondary antibodies with concentrations and suppliers is provided in the Supplementary Table S1. Counterstain DAPI was used to stain nuclei. Negative controls were prepared by excluding primary antibodies. Stained sections were imaged by using Fluorescence microscope (Zeiss). ImageJ was used for quantification of fluorescence (https://imagej.nih.gov/ij/)^87^.

Biochemical analysis of pro-inflammatory factors, IL1β, IL6, MCP1, and TNFα in the plasma was carried out by AssayGate, Inc., which uses Luminex multiplex bead assay platform for the analysis of multiple proteins.

### Data analysis

Results are expressed as Mean ± S.E.M. Number of experiments ‘n’ indicates the number of mice used per each treatment group. Treatments were compared for significant difference by unpaired ‘t’-test or by One-way ANOVA with Tukey’s post-test for multiple comparisons, as applicable. Some datasets were compared by Two-way ANOVA mixed-effects model with Bonferroni’s post-test for multiple comparisons. Prism software (version 8.12.0, GraphPad Software, Inc.) (www.graphpad.com) was used for carrying out statistical tests. Experimental groups were considered significantly different if P < 0.05.

## Supplementary Information

Supplementary Information.

## Data Availability

All datasets generated and analyzed for this study are included in this article. Access to raw files, where applicable, can be provided by the corresponding author upon request.
