# Supplementary material for: Reversal of aging-associated increase in myelopoiesis and expression of alarmins by angiotensin-(1–7)
Source: Sci Rep. 2023 Feb 13;13:2543. doi: 10.1038/s41598-023-29853-w (PMC9925828; doi:10.1038/s41598-023-29853-w)

Data Supplement

Reversal of Aging-Associated Increase in Myelopoiesis and Expression of Alarmins by Angiotensin-(1-7)

[1]Chittimalli K, [1]Jahan J, [1]Sakamuri A, [1]Weyrick H, [1]Winkle B, [2]Adkins S, [1]Vetter SW, [1ϕ]Jarajapu YP.

[1]Department of Pharmaceutical Sciences, College of Health Professions, North Dakota State University, Fargo ND 58108. [2]School of Biomedical Sciences, University of North Dakota, Grand Forks ND 58202.

Key words: Aging, Bone marrow, Angiotensin-(1-7), Ischemia, Myelopoiesis, monocyte-macrophages and Alarmins.

Running title: Angiotensin-(1-7) and myelopoiesis in aging

[ϕ]**Author for correspondence:**

Yagna PR Jarajapu, M Pharm, Ph D, FAHA.

Sudro-16, Albrecht Blvd.,

Department of Pharmaceutical Sciences,

College of Health Professions,

North Dakota State University,

Fargo, North Dakota, USA.

Email: Yagna.Jarajapu@ndsu.edu

Tel: (001) 701-231-8843

Fax: (001) 701-231-8333

Data Supplement

Supplementary Table 1. List of primary and secondary antibodies used for flow cytometry, western blotting and immunohistochemistry.

Target antigen	Vendor or Source	Catalog #	Working concentration	Persistent ID / URL
Lineage-depletion cocktail	Biolegend	133302	1 µL/100 µL	https://www.biolegend.com/en-gb/products/fitc-anti-mouse-lineage-cocktail-with-isotype-ctrl-5803?GroupID=BLG10793
7AAD	Biolegend	420404	0.3 µL/100 µL	https://www.biolegend.com/en-gb/products/7-aad-viability-staining-solution-1649
Ly6G	BD Biosciences	562700	0.125 µL/100 µL	https://www.bdbiosciences.com/en-us/products/reagents/flow-cytometry-reagents/research-reagents/single-color-antibodies-ruo/pe-cf594-rat-anti-mouse-ly-6g.562700
CD11b	Invitrogen	47-0112-82	0.06 µL/100 µL	https://www.thermofisher.com/antibody/product/CD11b-Antibody-clone-M1-70-Monoclonal/47-0112-82
Ly6C	BD Biosciences	553104	0.1 µL/100 µL	https://www.bdbiosciences.com/en-us/products/reagents/flow-cytometry-reagents/research-reagents/single-color-antibodies-ruo/fitc-rat-anti-mouse-ly-6c.553104
F4/80	Biolegend	123110	0.75 µL/100 µL	https://www.biolegend.com/en-gb/products/pe-anti-mouse-f4-80-antibody-4068
FPS-ZM1	Selleckchem	S8185	1µM	https://www.selleckchem.com/products/fps-zm1.html
CD31	Abcam	ab28364	1:50	https://www.abcam.com/CD31-antibody-ab28364.html
ILB4 - FITC	Enzo	ALX-650-001F-MC05	1:50	https://www.enzolifesciences.com/ALX-650-001F/isolectin-b4-bandeiraea-simplicifolia-fitc-conjugate/
DAPI	Biotium	40043	NA	https://biotium.com/product/dapi/
F4/80	Invitrogen	14-4801-82	1:50	https://www.thermofisher.com/antibody/product/F4-80-Antibody-clone-BM8-Monoclonal/14-4801-82
CD11b	Abcam	ab133357	1:4000	https://www.abcam.com/CD11b-antibody-EPR1344-ab133357.html
ACE	Thermo Fischer	MA5-32741	1: 1000	https://www.thermofisher.com/antibody/product/ACE-Antibody-clone-JM59-32-Recombinant-Monoclonal/MA5-32741
ACE-2	Thermo Fischer	MA5-32307	1: 1000	https://www.thermofisher.com/antibody/product/ACE2-Antibody-clone-SN0754-Recombinant-Monoclonal/MA5-32307
MAS1 (G-1)	Santacruz	Sc- 390453	1: 1000	https://www.scbt.com/p/mas1-antibody-g-1/
AT-1 (G-3)	Santacruz	Sc- 515884	1: 1000	https://www.scbt.com/p/at1-antibody-g-3?requestFrom=search
S100- A8	R & D Systems	MAB 3059	1: 1000	https://www.rndsystems.com/products/mouse-s100a8-antibody-335806_mab3059
S100- A9	R & D Systems	MAB 2065	1: 1000	https://www.rndsystems.com/products/mouse-s100a9-antibody-372510_mab2065
RAGE	Santacruz	Sc- 365154	1: 1000	https://www.scbt.com/p/rage-antibody-a-9/
HMGB-1	CST- cell signaling	3935	1: 1000	https://www.cellsignal.com/products/primary-antibodies/hmgb1-antibody/3935

Data Supplement

488 Goat Anti Rat	Invitrogen	A11006	1:100	https://www.thermofisher.com/antibody/product/Goat-anti-Rat-IgG-H-L-Cross-Adsorbed-Secondary-Antibody-Polyclonal/A-11006
633 Goat Anti Rabbit	Biotium	20122	1:250	https://biotium.com/product/goat-anti-rabbit-igg-hl/

Data Supplement

Supplementary Table 2: List of primer sequences that were used for determining the expression of different genes.

Description	Source / Repository	Concentration
NLRP1<br>F - 5'GGAGCCTTGTTCTTCAAAGACACA3'<br>R - 5'TTGATCAGAAGTGATAGAGGAGACC3'	Invitrogen	10 µM
NLRP3<br>F - 5'ACCAGCCAGAGTGGAATGAC-3'<br>R - 5' ATGGAGATGCGGGAGAGATA3'	Invitrogen	10 µM
NLRP4<br>F - 5'TACACAGCAGGAACGAAGACTCAG3'<br>R - 5'GGCTTCCACAGATGACCCACA3'	Invitrogen	10 µM
ASC<br>F - 5'AGTGGGCTGCTGGATGCTCTG3'<br>R - 5'CATCTTGCTTGGGTTGGTGG3'	Invitrogen	10 µM
AIM2<br>F - 5'AAGAGAGCCAGGGAAACTCC3'<br>R - 5'TGTCTCCTTCCTCGCACTT3'	Invitrogen	10 µM
IL-1β<br>F - 5'AGTTGACGGACCCCAAAAG3'<br>R - 5'CTTCTCCACAGCCACAATGA3'	Invitrogen	10 µM
IL-18<br>F - 5'ACAACTTTGGCCGACTTCAC3'<br>R - 5'GTCTGGTCTGGGGTTCACTG3'	Invitrogen	10 µM
CMA1<br>F - 5'CCTGGGTTCCAGCACCAA3'<br>R - 5'GGCGGGAGTGTGGTATGC3'	Invitrogen	10 µM
PU.1<br>F - GGGAGAGCCATAGCGACCAT3'<br>R - 5'TAGGAGACCTGGTGGCCAAGA3'	Invitrogen	10 µM
GATA1<br>F - 5′CACTCCCCAGTCTTTCAGGTGTA3′<br>R - 5′GGTGAGCCCCCAGGAATT3′	Invitrogen	10 µM
GATA2<br>F - 5′CACCTGTTGTGCAAATTGTCAGA3′<br>R - 5′GGATCCCTTCCTTCTTCATGGT3'	Invitrogen	10 µM
RUNX1<br>F - 5'TAGCGAGATTCAACGACCTC3'<br>R - 5'GTGGCGGATTTGTAAAGACG3'	Invitrogen	10 µM
RUNX2<br>F - 5'GTACTTCGTCAGCATCCTAT3'<br>R - 5'AGCGTGCTGCCATTCGAGGT3'	Invitrogen	10 µM
CCL2<br>F - 5'CCCAATGAGTAGGCTGGAGA3'<br>R - 5'AAAATGGATCCACACCTTGC3'	Invitrogen	10 µM
S100A1<br>F - 5'AATGTGTTCCATGCCCATTCG3'<br>R - 5'ACCAGCACAACATACTCCTTG3'	Invitrogen	10 µM
S100A2<br>F - 5'ACGCCAGTCAAGAGGACGA3'<br>R - 5'CCCCACATAGCTCAGCAGC3'	Invitrogen	10 µM
S100A3<br>F - 5'CAGTAGCTGCCATCGTGTG3'<br>R - 5'TACTCCCCAAAGTCCACTTCG3'	Invitrogen	10 µM

Data Supplement

S100A4<br>F - 5'TCCACAAATACTCAGGCAAAGAG3'<br>R - 5'GCAGCTCCCTGGTCAGTAG3'	Invitrogen	10 µM
S100A5<br>F - 5'ATTCAGGGAGAGAGGGTAGCA3'<br>R - 5'CTCTGCAAGACTCAGCTCTGT3'	Invitrogen	10 µM
S100A6<br>F - 5'GTCGACCGTGCGCTTCTTC3'<br>R - 5'TTCTTGCTCAGGGTGTGCTT3'	Invitrogen	10 µM
S100A7A<br>F - 5'TGCTCTTGGATAGTGTGCCTC3'<br>R - 5'GCTCTGTGATGTAGTATGGCTG3'	Invitrogen	10 µM
S100A8<br>F - 5'AAATCACCATGCCCTCTACAAG3'<br>R - 5'CCCACTTTTATCACCATCGCAA3'	Invitrogen	10 µM
S100A9<br>F - 5'ATACTCTAGGAAGGAAGGACACC3'<br>R - 5'TCCATGATGTCATTTATGAGGGC3'	Invitrogen	10 µM
S100A10<br>F - 5'TGGAAACCATGATGCTTACGTT3'<br>R - 5'GAAGCCCACTTTGCCATCTC3'	Invitrogen	10 µM
S100A11<br>F - 5'AAGTACAGCGGGAAGGATGGA3'<br>R - 5'ATGCGGTCAAGGACACCAG3'	Invitrogen	10 µM
S100A13<br>F - 5'AACTGCCTCATTTGCTCAAGG3'<br>R - 5'AGTCTCCAGTATTCACTGAACCT3'	Invitrogen	10 µM
S100A14<br>F - 5'ATGGGACAGTGTCGGTCAG3'<br>R - 5'CCGCCACAGAGTATTTATGGAAG3'	Invitrogen	10 µM
S100A16<br>F - 5'AAGTCCAGCTTCCGAAAGATG3'<br>R - 5'CCAGTATTCGTCAAAGCAGATGC3'	Invitrogen	10 µM
S100B<br>F - 5'TGGTTGCCCTCATTGATGTCT3'<br>R - 5'CCCATCCCCATCTTCGTCC3'	Invitrogen	10 µM
S100G<br>F - 5'ATGTGTGCTGAGAAGTCTCCT3'<br>R - 5'CGCCATTCTTATCCAGCTCCTT3'	Invitrogen	10 µM
S100P<br>F - 5'CGCACCATTTAATCCAACAGTTT3'<br>R - 5'GGTATTGGGACTGACCCCTTC3'	Invitrogen	10 µM
S100Z<br>F - 5'AGGAGGGCGACAGGTTCAA3'<br>R - 5'CCTTTTGGCATGTGAGGAACTC3'	Invitrogen	10 µM
HMGB<br>F - 5'GCTCAGAGAGGTGGAAGACCA3'<br>R - 5'GGTGCATTGGGATCCTTGAA3'	Invitrogen	10 µM
RAGE<br>F - 5'CTTGCTCTATGGGGAGCTGTA3'<br>R - 5'CATCGACAATTCCAGTGGCTG3'	Invitrogen	10 µM
β-actin<br>F - 5'CCATCATGAAGTCTGACGTTG3'<br>R - 5'CAATGATCTTGATCTTCATGGTG3'	Invitrogen	10 µM

Data Supplement

Figure legends

Supplementary Figure S1.

Gating strategy for monocyte-macrophage characterization by flow cytometry: Shown were representative flowcytometry dot plots for a sample of cells with isotype controls (upper panel) or antibodies (lower panel). Following exclusion of 7AAD-positive cells, sequential gating involved exclusion of doublets or cell clumps and selection of monocyte-lymphocyte gate for inflammatory cells. Neutrophils were excluded by selection of $Ly6G^-$ population. Monocyte-macrophages were selected by $CD11b^+$ population followed by $Ly6C^+$ to identify inflammatory monocytes or in combination with F4/80 to identify M1 ($Ly6C^{+hi}F4/80^{hi}$) and M2 macrophages ($Ly6C^{lo}F4/80^{lo}$).

Data Supplement

Supplementary Figure S1

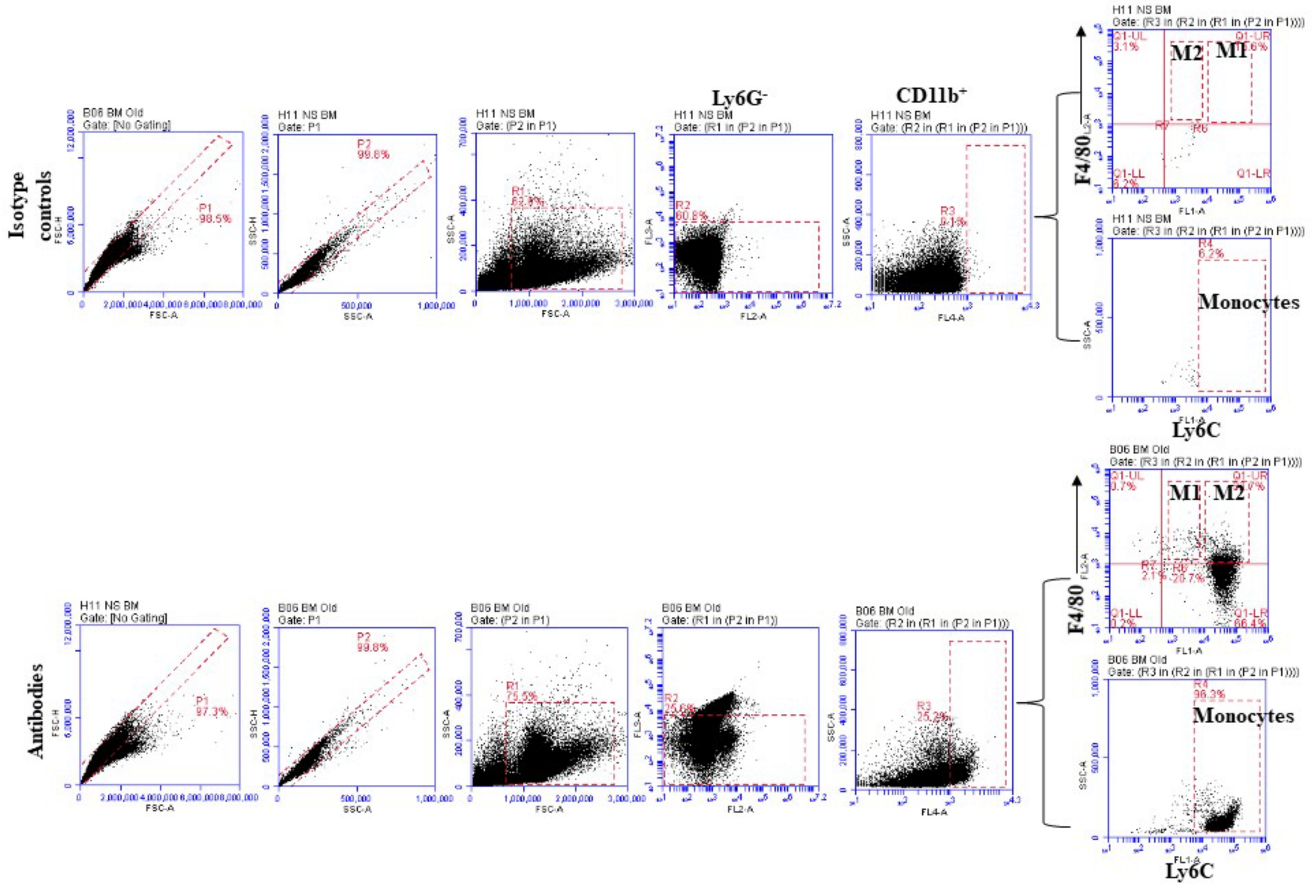

Original images of western blots

Figure 1A

MasR in the enriched BM-hematopoietic progenitors

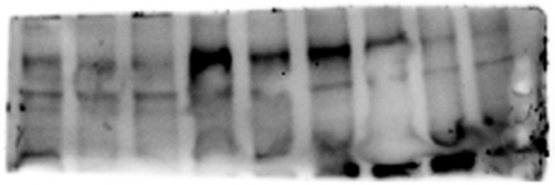

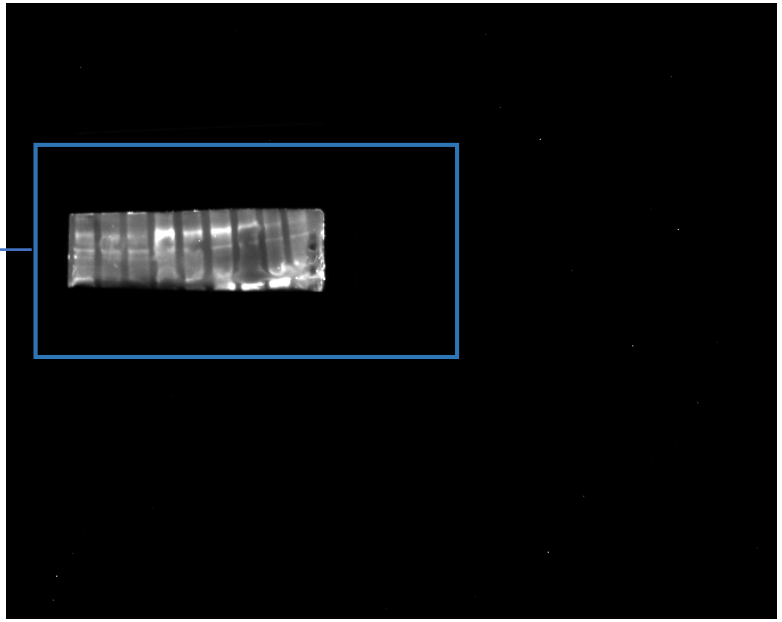

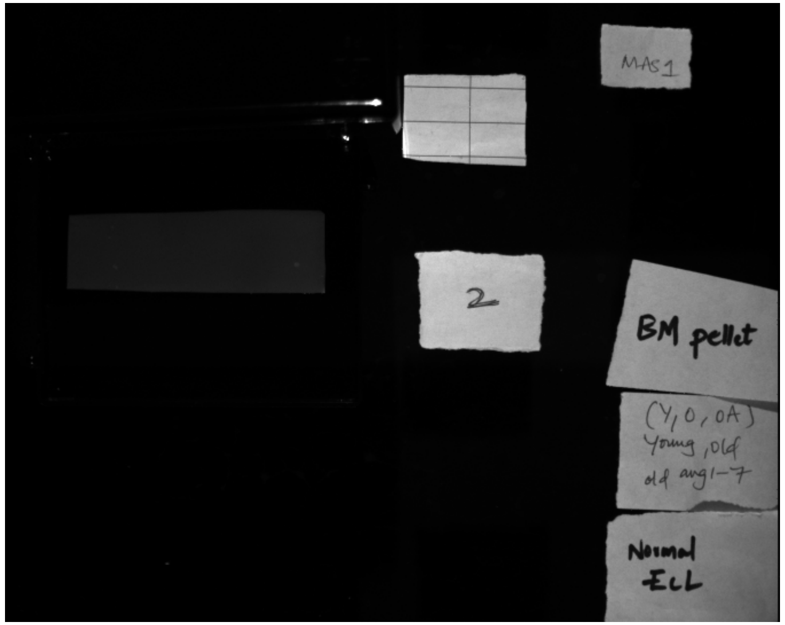

Figure 1A

AT1R in the enriched BM-hematopoietic progenitors

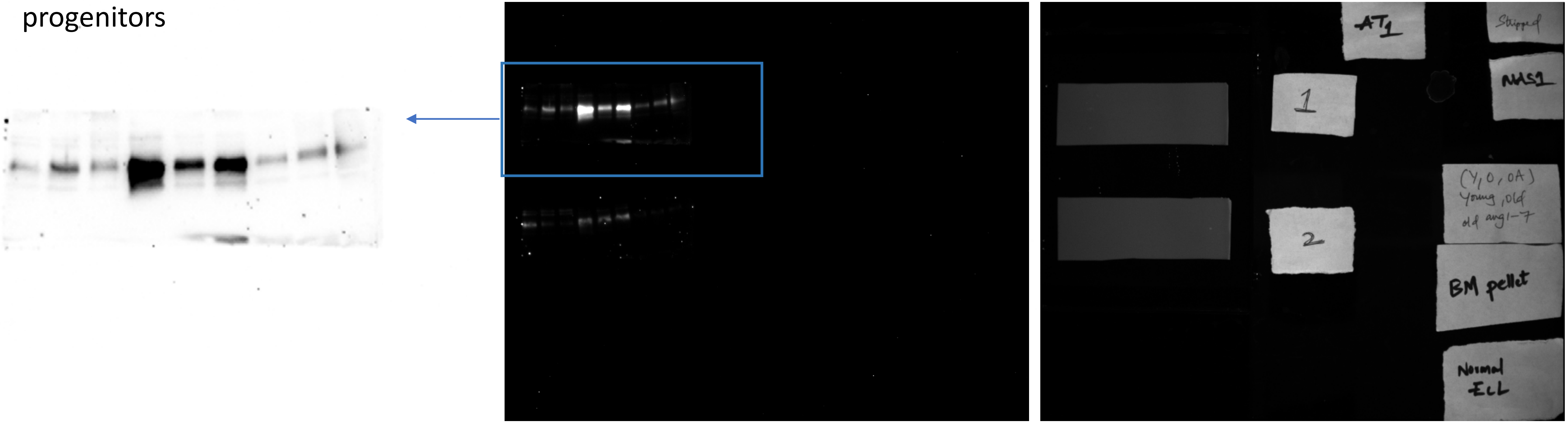

Figure 1A

β-actin in the enriched BM-hematopoietic progenitors

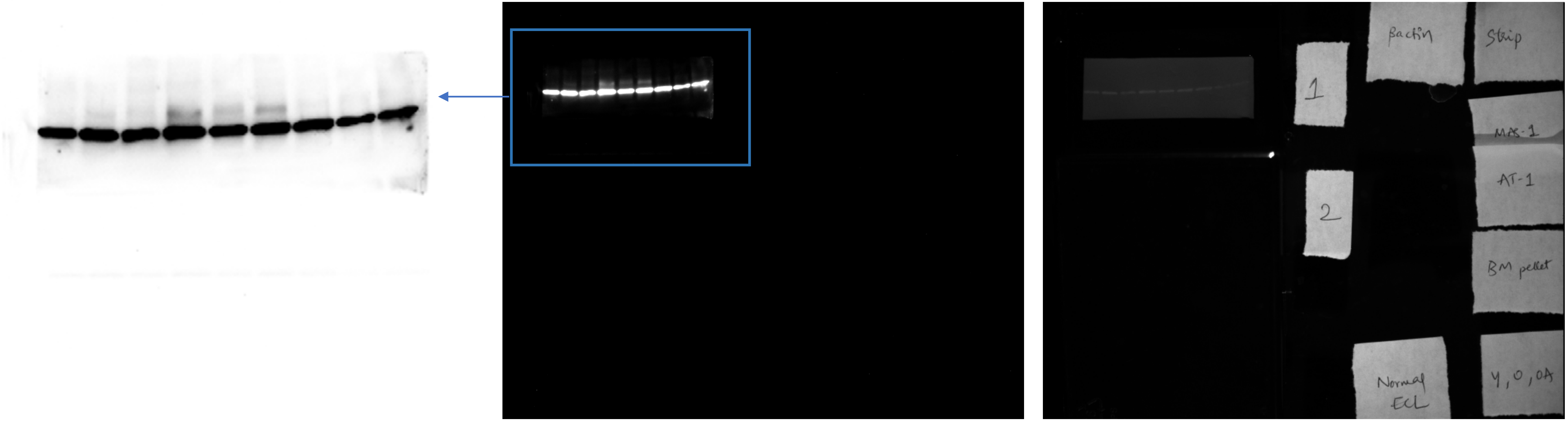

Figure 1C

ACE in bone marrow supernatants

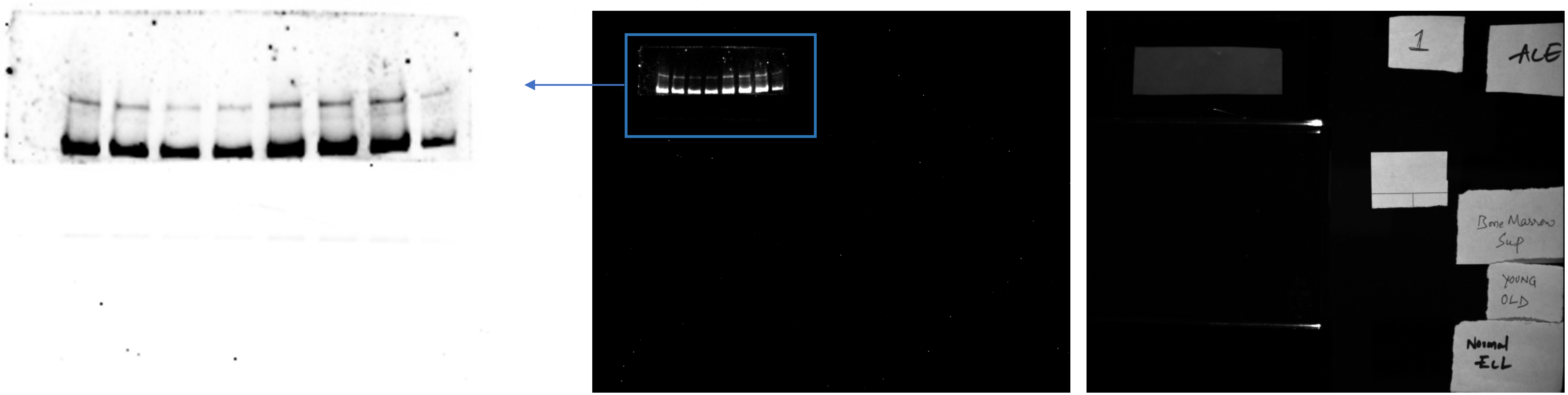

Figure 1C

ACE2 in bone marrow supernatants

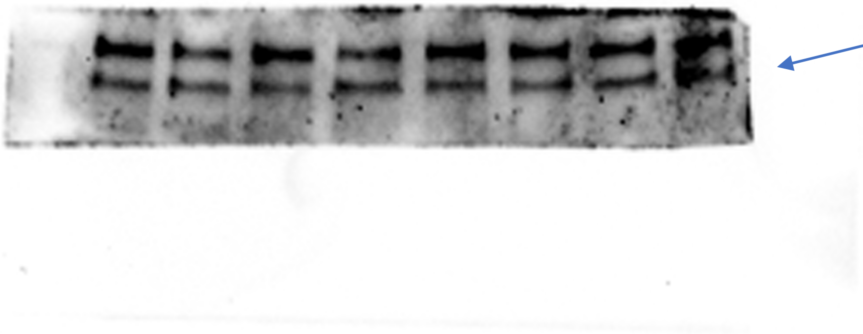

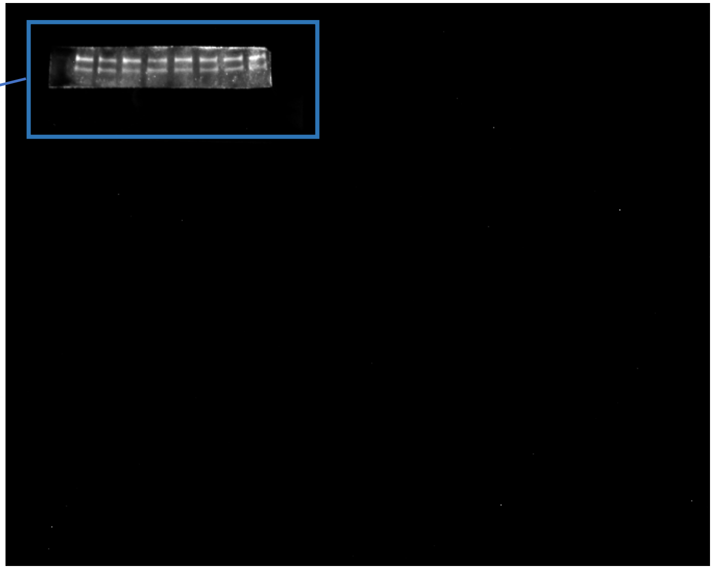

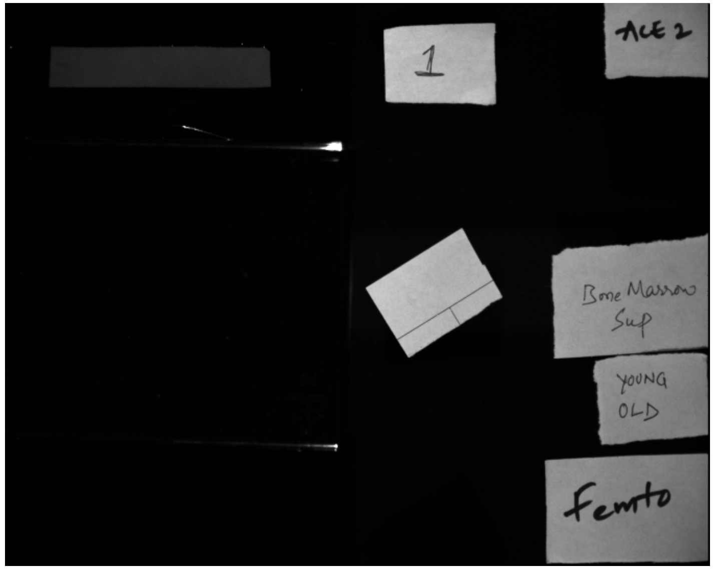

Ponceau S in BM Supernatants

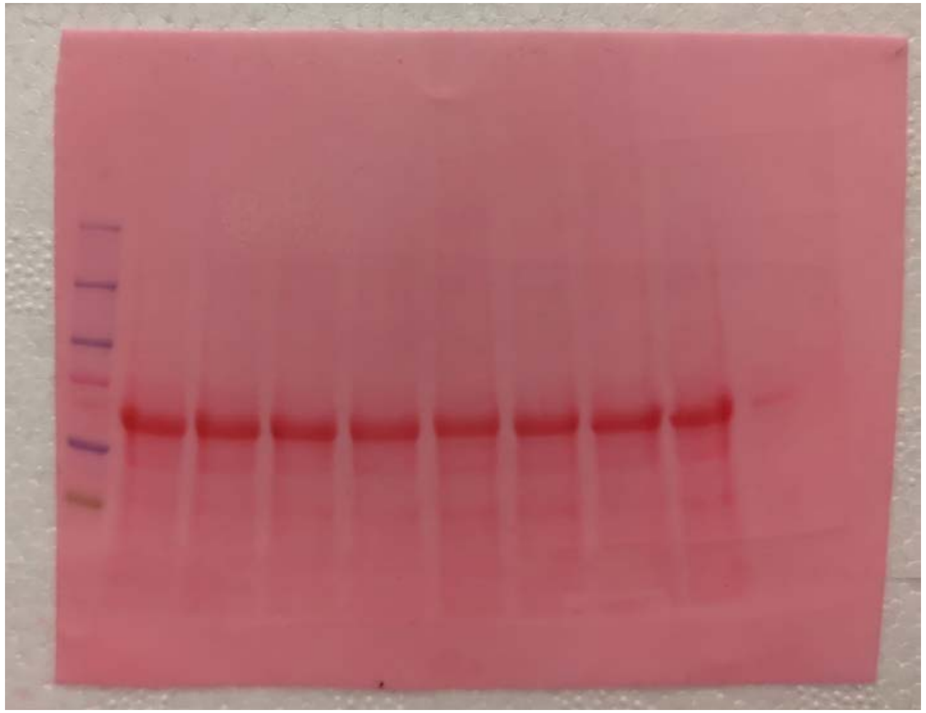

Figure 6A

S100A8/A9 in BM supernatants

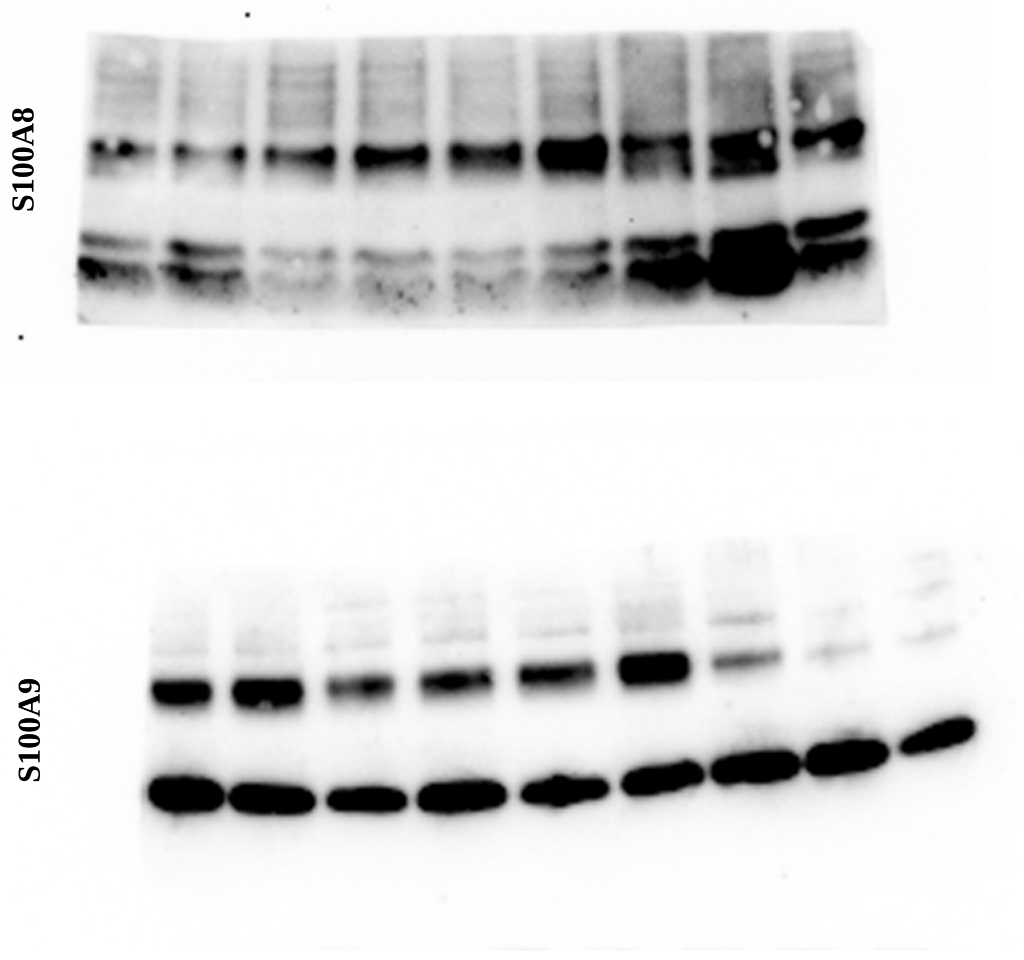

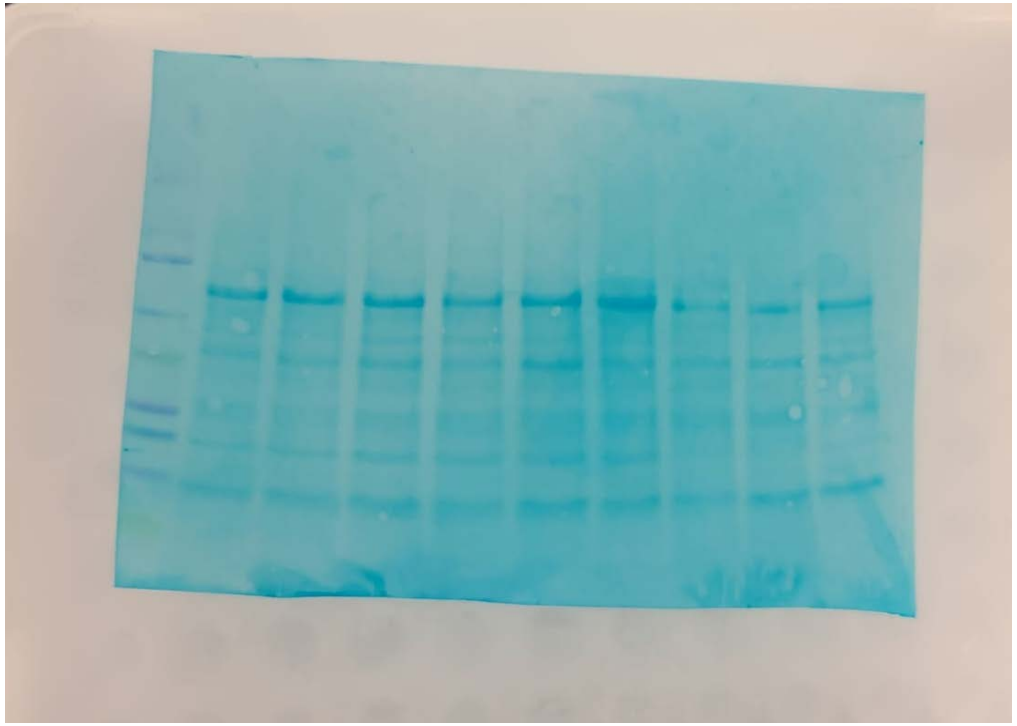

Figure 6A

S100A8 in BM supernatants

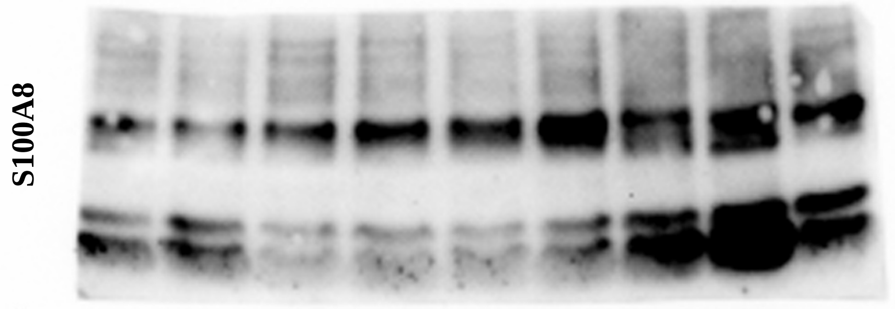

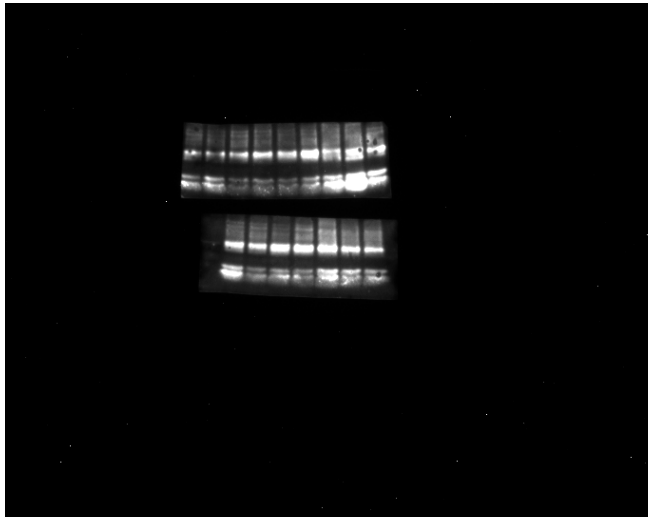

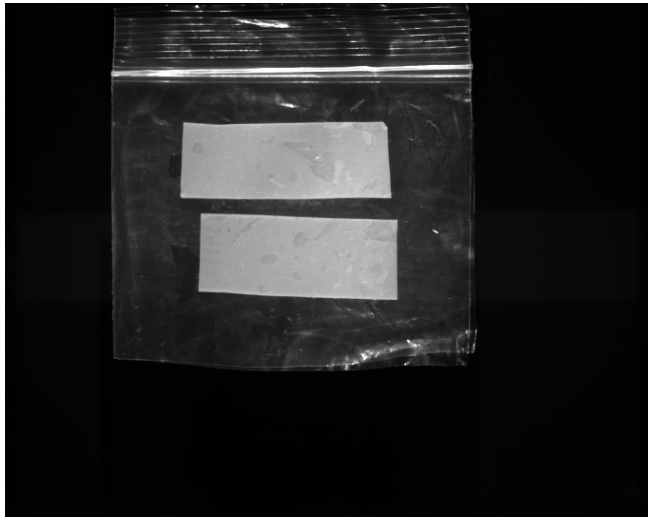

Figure 6A

S100A9 in BM supernatants

S100A9

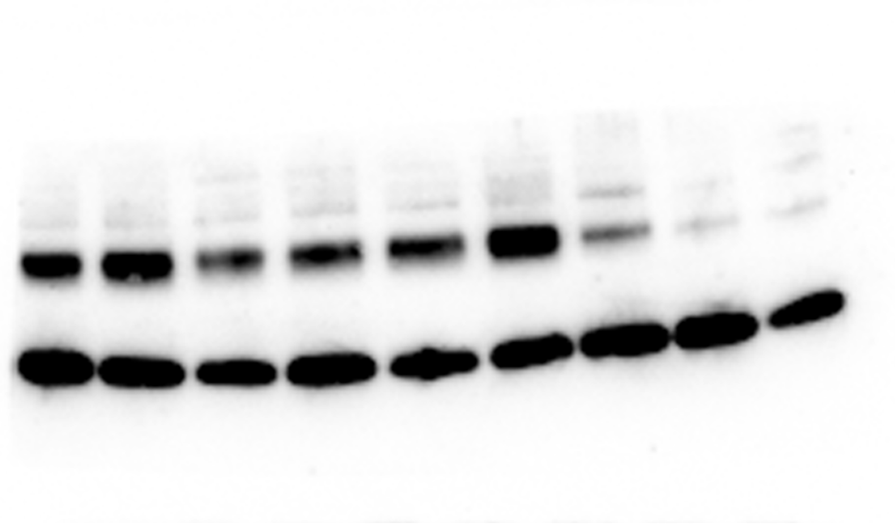

Memcode

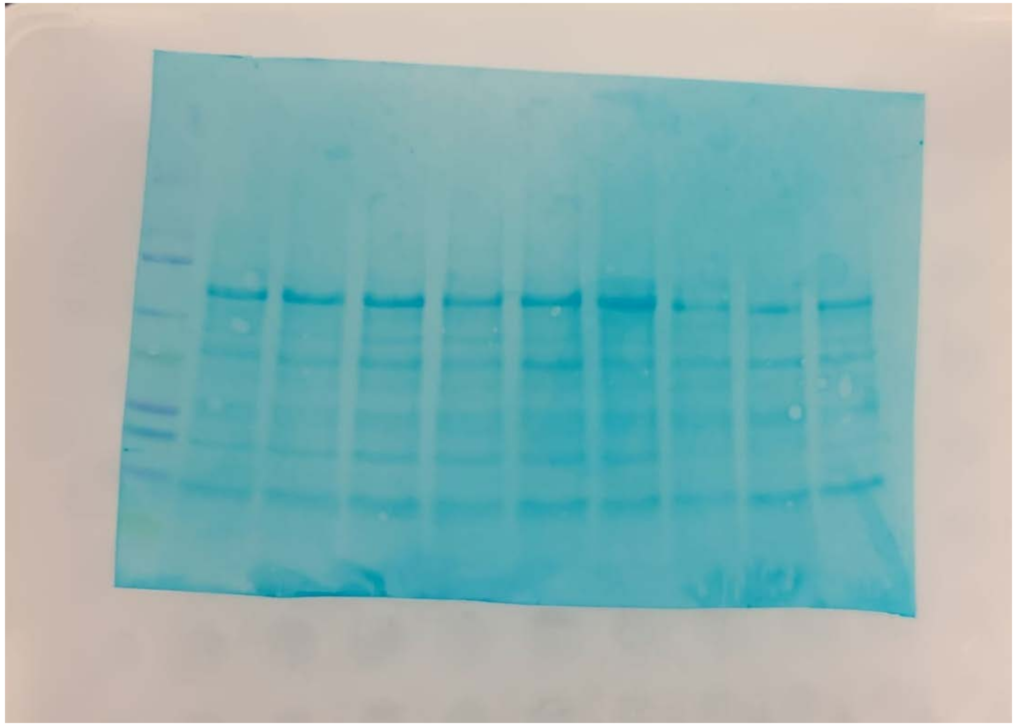

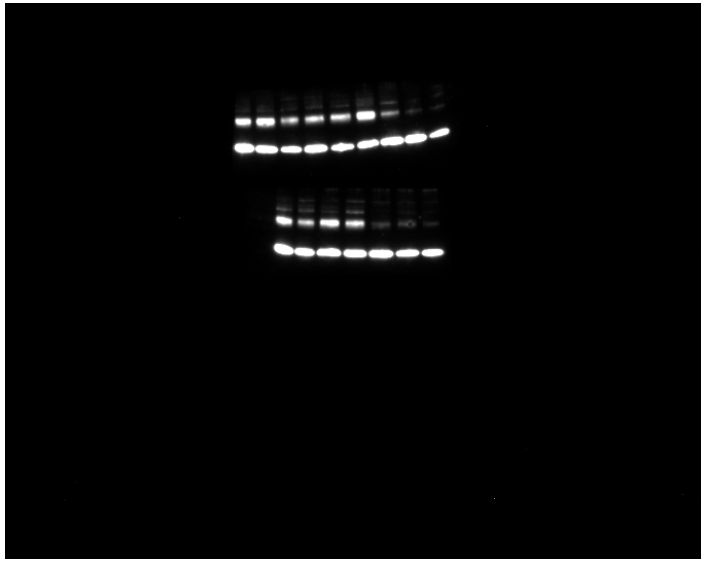

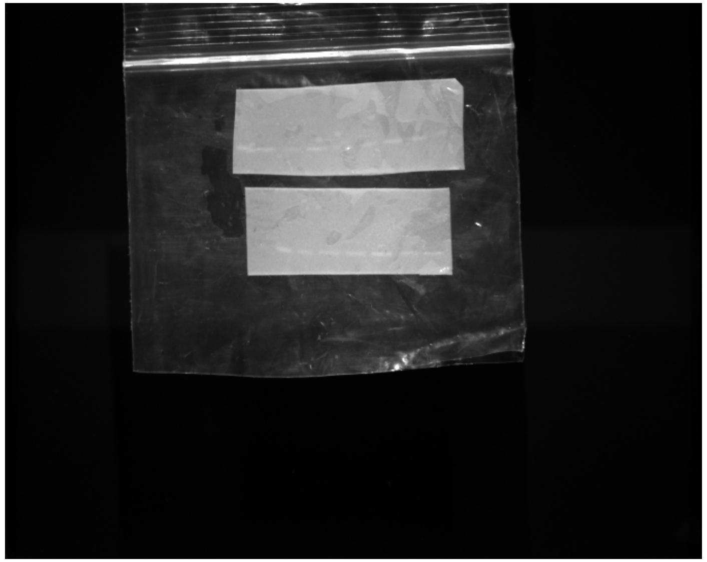

Figure 6D

HMGB1 in BM supernatants

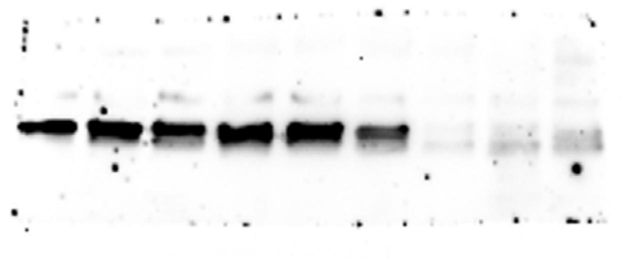

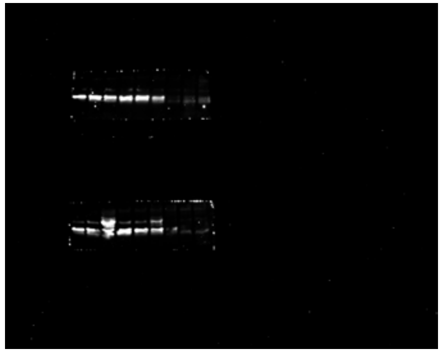

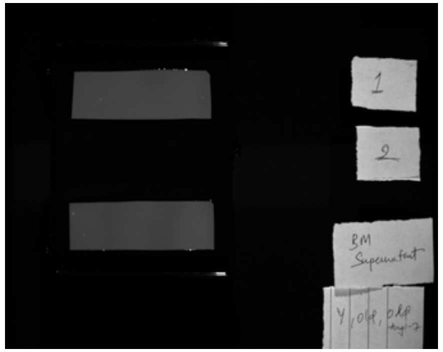

Ponceau S

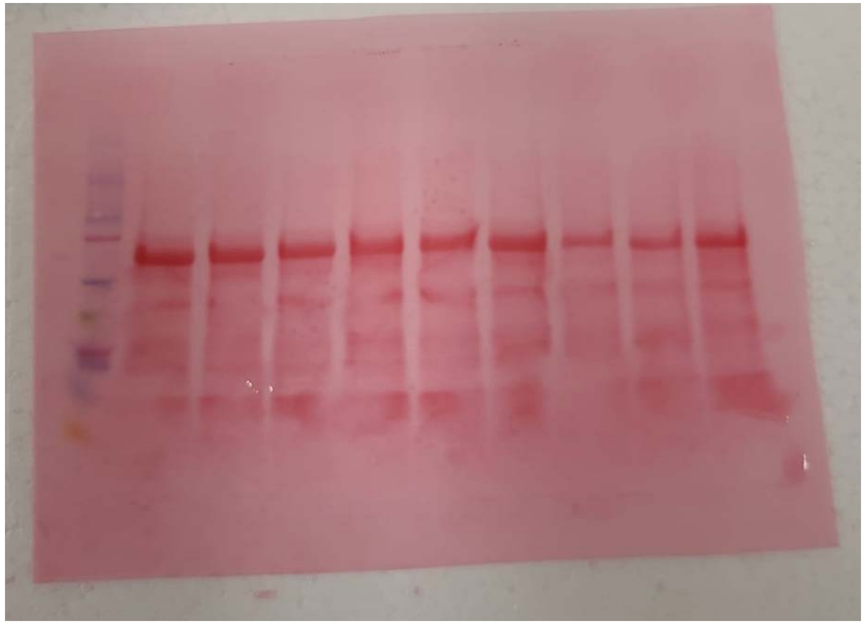

Figure 6F

RAGE in enriched BM-hematopoietic progenitors

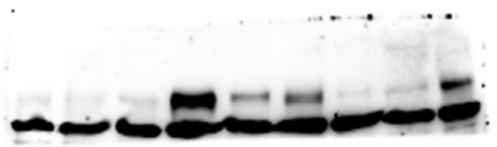

Beta actin

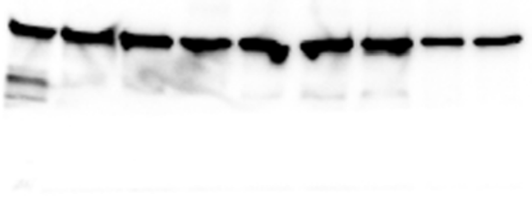

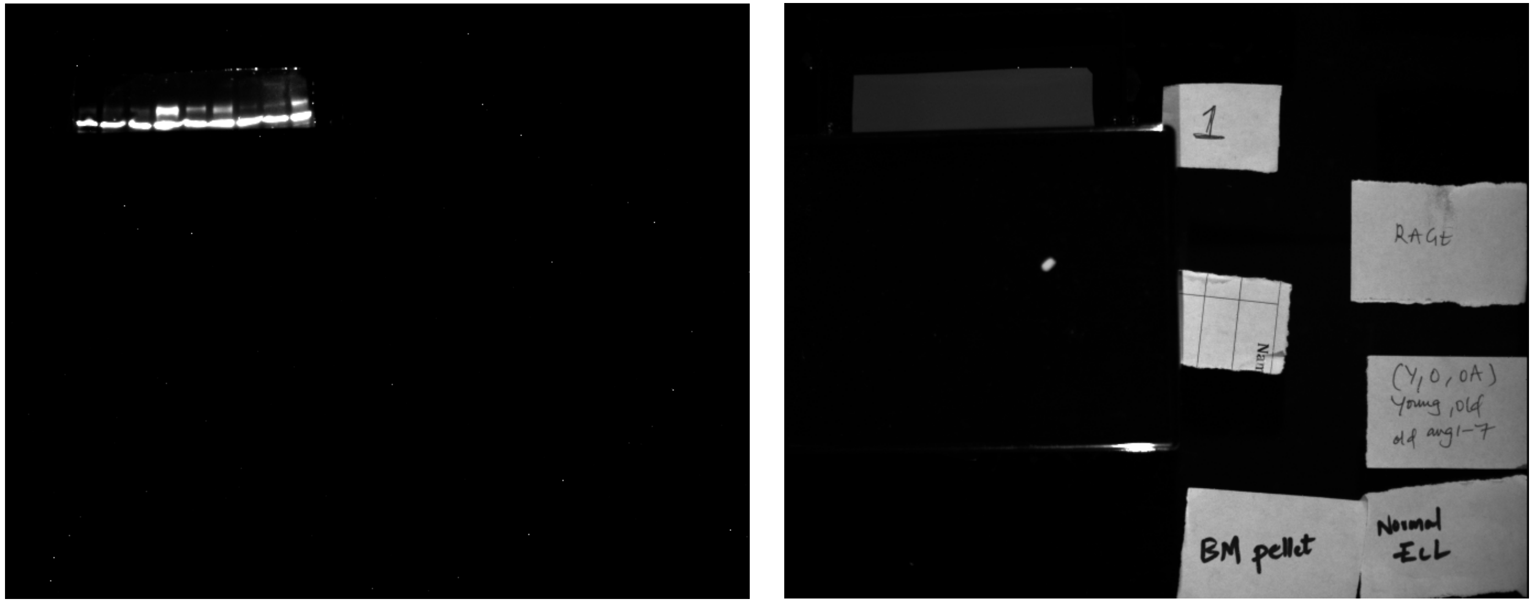

Figure 7A

HMGB1 in plasma

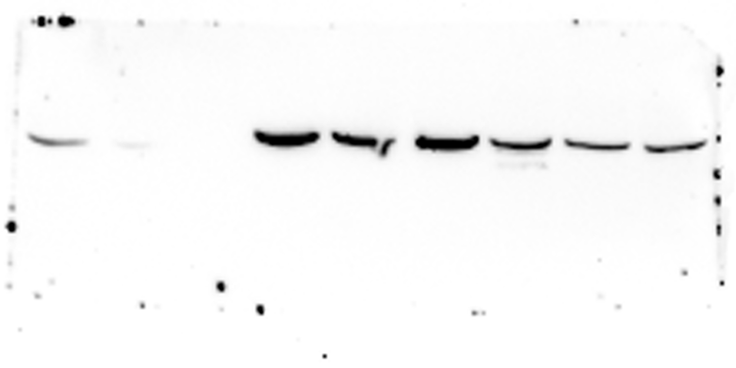

Ponceau S plasma

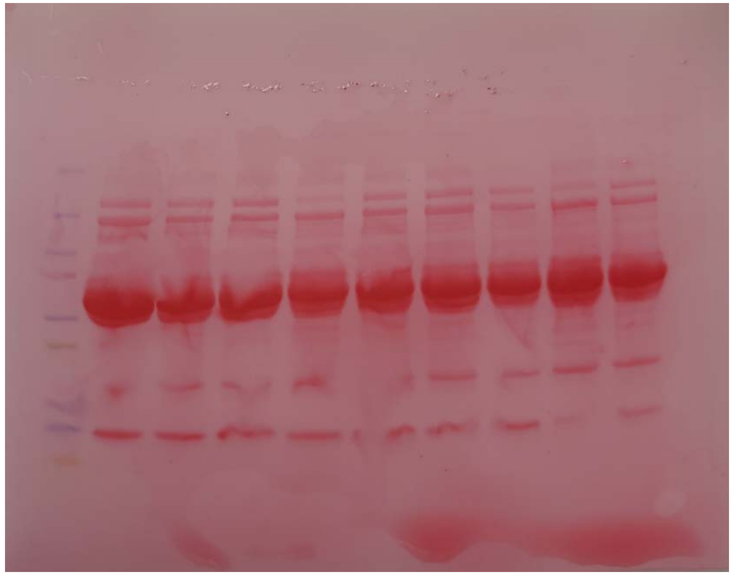

Supplement: Supplementary file 1 — Supplementary Information. [file 41598_2023_29853_MOESM1_ESM.pdf]
